# Genomic regions involved in the control of 1,000-kernel weight in wild relative-derived populations of durum wheat

**DOI:** 10.3389/fpls.2023.1297131

**Published:** 2023-11-30

**Authors:** Yaman Jabbour, Mohammad Shafik Hakim, Abdallah Al-Yossef, Maysoun M. Saleh, Ahmad Shams Al-Dien Shaaban, Hafssa Kabbaj, Meryem Zaïm, Charles Kleinerman, Filippo M. Bassi

**Affiliations:** ^1^ Field Crop Department, Faculty of Agriculture Engineering, Aleppo University, Aleppo, Syria; ^2^ General Commission for Scientific Agriculture Research (GCSAR), Field Crop Department, Aleppo, Syria; ^3^ General Commission for Scientific Agriculture Research (GCSAR), Genetic Resources Department, Damascus, Syria; ^4^ Biotechnology Engineering Department, Faculty of Technological Engineering, Aleppo University, Aleppo, Syria; ^5^ International Center for Agricultural Research in the Dry Areas, Biodiversity and Crop Improvement, Rabat, Morocco

**Keywords:** wild relatives, drought, nested association mapping NAM, grain size, TKW

## Abstract

Terminal drought is one of the most common and devastating climatic stress factors affecting durum wheat (*Triticum durum* Desf.) production worldwide. The wild relatives of this crop are deemed a vast potential source of useful alleles to adapt to this stress. A nested association mapping (NAM) panel was generated using as a recurrent parent the Moroccan variety ‘Nachit’ derived from *Triticum dicoccoides* and known for its large grain size. This was recombined to three top-performing lines derived from *T. dicoccoides*, *T. araraticum*, and *Aegilops speltoides*, for a total of 426 inbred progenies. This NAM was evaluated across eight environments (Syria, Lebanon, and Morocco) experiencing different degrees of terminal moisture stress over two crop seasons. Our results showed that drought stress caused on average 41% loss in yield and that 1,000-kernel weight (TKW) was the most important trait for adaptation to it. Genotyping with the 25K TraitGenetics array resulted in a consensus map of 1,678 polymorphic SNPs, spanning 1,723 cM aligned to the reference ‘Svevo’ genome assembly. Kinship distinguished the progenies in three clades matching the parent of origin. A total of 18 stable quantitative trait loci (QTLs) were identified as controlling various traits but independent from flowering time. The most significant genomic regions were named Q.ICD.NAM-04, Q.ICD.NAM-14, and Q.ICD.NAM-16. Allelic investigation in a second germplasm panel confirmed that carrying the positive allele at all three loci produced an average TKW advantage of 25.6% when field-tested under drought conditions. The underlying SNPs were converted to Kompetitive Allele-Specific PCR (KASP) markers and successfully validated in a third germplasm set, where they explained up to 19% of phenotypic variation for TKW under moisture stress. These findings confirm the identification of critical loci for drought adaptation derived from wild relatives that can now be readily exploited via molecular breeding.

## Introduction

Durum wheat (*Triticum durum* Desf.) is one of the most important cereal crops worldwide (Gonzalez-Segura et al., 2014) and one of the central pillars of global food security in the Mediterranean region (Graziano et al., 2020) where more than 50% of durum wheat production is concentrated (Sall et al., 2019). Along the Mediterranean, cultivation is mostly conducted in rainfed conditions with both drought and heat stress frequently occurring during the grain filling stage (Palta and Turner, 2018; Xynias et al., 2020) causing a severe reduction in grain yield (Slafer et al., 2005; Daryanto et al., 2016; Sarto et al., 2017). In Syria, the drought of the 2008–2009 season resulted in a 50% reduction of durum wheat production and 45% in the dry season of 2013–2014 and up to 63% in the dry season of 2017–2018 (Annual Agriculture Statistical Abstract, 2020). Similarly, Morocco experienced a 50% reduction in total durum wheat production in the extremely dry season of 2021–2022 (United States Department of Agriculture, Foreign Agricultural Service, 2022).

Maintaining farms’ productivity despite severe drought stress requires the deployment of tolerant varieties (Van Oosten et al., 2016). However, the narrow genetic base of many durum wheat breeding programs (Jing et al., 2013; Bassi and Nachit, 2019; Mazzucotelli et al., 2020) has led to a reduction in allelic diversity, which reduces the chances of developing novel germplasm capable of adapting to these stresses (Makai et al., 2016). Thus, many authors suggest the integration of wider germplasm such as landraces, primitive wheat, and crop wild relatives (CWRs) into the breeding process to increase useful genetic variance (Reynolds et al., 2018; Ledesma-Ramírez et al., 2019). CWRs have survived for millennia in harsh environments without the help of human farmers and should hence represent true treasure troves of useful alleles (Kiani et al., 2015; Masoomi-Aladizgeh et al., 2015; Zhang et al., 2016; El Haddad et al., 2020). The usefulness of primitive wheat and CWR for durum wheat is well documented as a source of resistance to all major biotic (Marais et al., 2005; Marais et al., 2010; Babaiants et al., 2012; Bassi et al., 2019; El Haddad et al., 2020) and abiotic stresses (Sall et al., 2018; Djanaguiraman et al., 2019). A fitting example is the Moroccan cultivar ‘Nachit’ derived from *T. dicoccoides*, which achieved higher yield potential and grain size when grown under drought conditions (Taghouti et al., 2023). Aberkane et al. (2021) also revealed that durum genotypes derived from *T. urartu* and *T. dicoccoides* were the top performers under drought stress. Similarly, El Haddad et al. (2021) confirmed that integrating CWR into durum wheat elite lines improved their overall performances: elites derived from *T. dicoccoides* and *Aegilops speltoides* were more tolerant to climatic stresses, while *T. araraticum-*derived lines had better pasta firmness and bread-making quality.

Despite the overall improvement that CWR provided, tagging with molecular markers their most useful alleles remains the most strategic method to easily transfer CWR-positive traits into modern cultivars. Mapping populations (MP) for QTL discovery and germplasm panels for genome-wide association studies (GWAS) are ideal methods to associate alleles to the trait they influence. However, when seeking the identification of CWR-derived alleles, some additional considerations are needed. In fact, a CWR allele is by definition “rare” and cannot be effectively studied by GWAS unless multiple lines of the panel carry it. It is instead possible to identify it by MP, but this approach typically suffers from a limited ability to transfer the discovery to other genetic backgrounds. Hence, the use of combined multiple mapping approaches has become the method of choice to identify CWR alleles (Scott et al., 2020). In particular, the nested association mapping (NAM) approach relies on one (or more) recurrent parent that is recombined into several “donor” parents. The result is a balanced panel of progenies with several “donor” alleles contrasting the “recurrent” allele so that the effects of both can be identified via QTL analysis and GWAS (Alahmad et al., 2020; Alahmad et al., 2022; Chidzanga et al., 2022).

In this study, we developed a large NAM of top-yielding durum wheat elites derived from CWR and tested it across eight environments experiencing contrasting levels of terminal moisture stress. The most stable and top-performing entries were defined using AMMI wide adaptation index’ (AWAI). Ultimately, the genomic regions associated with the tolerance were identified by GWAS and QTL analysis, then confirmed by allelic investigation in a second germplasm panel, and finally validated to Kompetitive Allele-Specific PCR (KASP) in a third panel.

## Materials and methods

### Plant material

#### Nested association mapping (NAM) panel 1

A durum wheat NAM of 426 F_5_-derived recombinant inbred lines (RILs) was obtained by crossing to the same recurrent parent three top-performing donor lines originating from the International Center for Agricultural Research in Dry Areas (ICARDA) to produce three subpopulations (**Table S1**). The recurrent parent was ‘Nachit’ (Amedakul1/TdicoSyrCol//Loukos), a Moroccan variety released in 2017, generated by a top cross of two elites to *T. dicoccoides* collected from Syria (Taghouti et al., 2023). ‘Nachit’ was selected as a recurrent parent because of its deep root system suitable to tolerate terminal drought (El Hassouni et al., 2018), early flowering, and very large grain size (Taghouti et al., 2023). The first donor parent was the elite ‘DAWRYT110’ (Amedakul1/TdicoSyrCol//Cham1) derived from a top cross with *T. dicoccoides* and deemed tolerant to terminal drought (El Haddad et al., 2021), contributing 146 RILs. The second donor parent was ‘Faraj’ (*T. araraticum* F4/3/Arthur71/Lahn//Blk2/Lahn/4/Quarmal), which is a Moroccan cultivar released in 2007 for its adaptation to low moisture conditions, characterized by delayed flowering and carrying a *T. araraticum* insertion that ensures resistance to Hessian fly (Bassi et al., 2019), contributing 138 RILs. The third donor was ‘Jabal’ (Korifla/AegspeltoidesSyr//Mrb5) derived from a top cross with *Ae. speltoides*, released in Morocco in 2021 for its shallow root system well adapted to the Atlas Mountains rocky soils (El Haddad et al., 2020), contributing 142 RILs.

#### Genome-wide association study panel 2

A second panel named the “GWAS panel” was investigated to confirm the NAM discoveries. The panel was described in detail by Kabbaj et al. (2017). Briefly, it comprises 96 landraces from 24 countries and 288 cultivars and elite breeding lines from eight countries. A total of 10 subpopulations have been identified within it (Kabbaj et al., 2017), and it has already been successfully used to identify the genomic loci involved in resistance to a damaging insect pest (Bassi et al., 2019), phenology (Gupta et al., 2020), and its responses to heat stress (El Hassouni et al., 2019) and moisture stress (Zaïm et al., 2023). This panel includes the parents of the NAM populations and their sister lines.

#### Marker-assisted selection validation panel 3

A third panel defined as the “marker**-**assisted selection (MAS) validation panel” was used to further validate the discovery. It includes 80 of ICARDA’s elites that constituted the 2020 International Nurseries 43rd IDON. These elites were derived by breeding selection after recombining the recurrent and donor parents with other elite lines (**Table S2**).

### Field trials

For the NAM panel, field trials were conducted during the two growing seasons 2018/2019 (19) and 2019/2020 (20), in eight different agroclimatic conditions (environments) across three countries (Syria, Lebanon, and Morocco) as described in **Table S3**. During season 19, planting was done in Lebanon under rainfed conditions at the station of Kfardan located 20 km east of Baalbek (34°01′01.2″N, 36°04′02.6″E) with an elevation of 1,100 m above sea level and in Syria at the station of Alsefera located 25 km southeast of Aleppo (36°04′00″N, 37°22′00″E) with an elevation of 348 m above sea level under rainfed conditions and at the station of Hemiama located 56 km east of Aleppo (36°09′N, 37°42′E) with an elevation of 356 m above sea level under supplemental irrigation. In season 20, planting occurred under rainfed and supplementary irrigated conditions in Lebanon at the station of Kfardan and in Syria at the station of Hemiama, while in Morocco, planting occurred under rainfed conditions only at the station of Marchouch (333403.10 0 N and 63800.10 0 W) with an elevation of 421 m above sea level. A total of three environments (Syr19, Syr20, and Leb20) received supplemental irrigation to avoid the occurrence of terminal drought and generate contrasting phenotypic effects. In Syr19, two gravity irrigations of 20 mm each 2 weeks apart were provided after flowering (stage of Zadok’s scale 65); in Syr20, one gravity irrigation of 20 mm was provided at flowering time (Z65) and two additional ones of 20 mm each were provided 2 weeks apart; in Leb20, one-sprinkle irrigation of 20 mm was provided 4 weeks after flowering. The remaining five environments (Leb19, Leb20, Mor20, Syr19, and Syr20) were rainfed and exposed the germplasm to severe terminal drought during the grain filling stage, with the following cumulative rainfalls after flowering: 8, 20, 18, 13, and 10 total mm, respectively. All moisture details are presented in **Table S4**.

Daily climatic information was collected from weather stations located at each test site. A climate matrix with maximum, minimum, and average temperatures and total moisture was calculated for four major growth stages for each environment: 1 month before sowing till sowing, sowing until the end of the vegetative stage (Z00–Z58), flowering stage (Z60–Z69), and grain filling period between flowering and maturity (Z71–Z92) as described in **Table S4**. The climate variables were then regressed against the average value at each location for GY and TKW. Significant interactions identified the climate factors influencing these traits. The significant climatic factors were then used to cluster the environments using Ward’s method based on Euclidean distance via *dendextend* R package (Galili and Dendextend, 2015). Two environmental clusters were determined as drought-affected and not affected, and combined analysis across environments was performed for each using ANOVA setting environments and genotypes as random factors.

Agronomic practices varied based on the location but followed the general guidelines of timely sowing between the 15th of November and the 15th of December, with 50 kg ha^−1^ of phosphorus, nitrogen, and potassium provided as base fertilization before planting and a total of 100 kg ha^−1^ of nitrogen provided in two equal split applications, the first one 4 weeks after emergence and the second before booting (Z45). One tank mixture of herbicide was applied before flowering to provide protection against monocots and dicots, and it was followed by mechanical weeding as needed to ensure clean plots.

In all sites, the accessions and the four parents were planted in a partially replicated (augmented) design of 10 blocks of size 48 including again in each block the four parents (Nachit, DAWRYT110, Faraj, Jabal) as replicated checks. The plot planting surface was of 1.5 m^2^ at a sowing density of 130 kg ha^−1^.

The GWAS panel was tested as presented in detail by Gupta et al. (2020) using plots of size 3 m^2^ and a partially replicated (augmented) design of 19 blocks each with four replicated checks. A total of 13 environments were used to define the phenological variation by Gupta et al. (2020). Among these, the results collected for 1,000-kernel size (TKW) were obtained from five environments experiencing terminal drought stress. The BLUEs of these individual environmental values were combined into one by expressing the performance of each entry as the average of the ratio to the best entry at each site.

The MAS validation panel is an international nursery of ICARDA, and as such, it was planted across 42 international locations by partners (Bassi and Sanchez-Garcia, 2017). Among these locations, seven environments experienced terminal drought stress. As per the GWAS panel, the best linear unbiased estimators for the TKW results from these individual environments were combined into one value by expressing the performance of each entry as the average of the ratio to the best entry at each site.

### Phenotypic data recording

The following traits were measured for each plot. Days from germination to the time of heading (DtH) was determined when 50% of the plot showed spikes emerging from the flag leaf (Z59) as a proxy to measure flowering time, which is harder to measure correctly in the autogamous and cleistogamous wheat flower. Days to maturity (DtM) was defined as the number of days from the germination date to the date when 50% of the spikes turned yellow (Z91–92). The grain filling period (GFP) was defined as the number of days elapsing between DtH and DtM. Plant height (PLH) was measured in cm from the ground to the top of a representative ear excluding its awns. Spikes per meter square (Spk.m^2^) was defined as the number of fertile spikes per linear meter, counted in one row of the plot, and then multiplied by 4 to obtain the number per m^2^. Grain yield (GY) was measured as the weight of the grains harvested from the plot expressed in kg, divided by the number of m^2^ harvested for the plot, and then multiplied by 10,000 m^2^ to obtain yield in kg ha^−1^. Thousand kernel weight (TKW) was obtained by weighting 200 random kernels and multiplying the value by 5. Grain filling rate (GFR) defines the speed at which a given genotype fills its grains, and it is calculated as TKW divided by GFP. The number of grains per meter square (Gr.m^2^) was calculated using the weight of the grains harvested from a 1.5-m^2^ area and the average weight of one kernel derived from the TKW value, as per:


$$Gr.m^{2}=\;\frac{Harvested\;weight\;of\;plot}{1.5m^{2}x\;\frac{TKW}{1000}}$$

The number of grains per spike (Grn.Spk) was calculated by dividing the number of grains per unit area by the number of spikes recorded for the same area, as follows:


$$Grn.spk=\;\frac{\;Gr.m^{2}}{Spk.m^{2}}$$

### Phenotypic data analysis

Spatial analysis was used to correct field differences using the row and column design by the *statgenSTA* package of R version 3.2.1 (R Core Team, 2017) and obtain the best linear unbiased predictors (BLUPs) for each environment of each trait. The single environment performances were then combined for each of the two climatic clusters (drought-stressed and non-drought-stressed) and across all environments using the package *agricolae* (R Core Team, 2017). Heritability was calculated based on the modified method suggested by Burton and Devane (1953). The phenotypic correlation between GY and all the other traits was computed using Multi Environment Trial Analysis with R for Windows (META R) (Alvarado et al., 2015), while the path analysis used R version 3.2.1 with the *agricolae* package (R Core Team, 2017). The “AMMI wide adaptation index” (AWAI) for GY and TKW was calculated using the following formula:


$$AWAI=\;\sum_{i}s_{i}\;*\;absolute\;value\;for\;IPCA_{i}$$

where *i* is the number of significant interaction principal components axes (IPCAs) for the AMMI and *s_i_* is the percentage of total G×E variation explained by each IPCA. AWAI values close to “0” are obtained for the most widely adapted germplasm (Bassi and Sanchez-Garcia, 2017). In order to determine the best genotypes combining both GY potential and stability, a biplot was drawn between the BLUP and AWAI scores.

### Molecular analysis of NAM panel 1

The NAM population was genotyped using the 25K TraitGenetics array (GmbH, Gatersleben, Germany), which combines the most polymorphic markers from the Axiom Breeder Array and the Illumina Infinium 90K (Mulugeta et al., 2023). High-fidelity polymorphic SNPs were defined as those having less than 1% missing data and a minor allele frequency superior to 10% as can be expected in a biparental population. All SNPs were aligned to the Svevo genome assembly (Maccaferri et al., 2019). Three linkage maps were generated (one for each subpopulation) as described in **Table S1** using QTL IciMapping V4.2 (Meng et al., 2015), assigning markers to different linkage groups through the “MAP” function at LOD of 5, and then ordering them at LOD of 3 using the “by anchor order” algorithm to respect their physical position. The Kosambi mapping function was then used to generate the three individual maps. These were combined into a consensus map using the CMP function: first by regrouping markers at a distance of less than 20 cM to obtain one group for each chromosome, then using the “by anchor order option” to measure the genetic distances between markers along the consensus map based on their relative positions on each individual map.

Linkage disequilibrium (LD) decay was estimated using the Neanderthal method (Jujumaan, 2017) for the consensus map using *r* ^2 ^= 0.2 as the threshold for significant linkage. The genetic structure of the populations was evaluated using the Bayesian clustering algorithm in Structure 2.3.2 software (Pritchard et al., 2000). A neighbor-joining tree was generated by calculating the genetic distance defined by Power Marker 3.25 (Liu and Muse, 2005) as calculated by Nei et al. (1983), and then the result was imported to MEGA V.10.2.5 software for analysis (Kumar et al., 2018).

Marker-trait associations (MTAs) were searched for the following phenotypic combinations: all traits’ BLUP value for each genotype in each environment, the combined value for the drought-stressed cluster, the combined value for the not stressed cluster, and combined across all environments. For GY and TKW, the investigation included the AWAI value. Associations were searched by QTL mapping for each individual subpopulation using the BIP function by ICIM-ADD in IciMapping V4.2 software (Meng et al., 2015). The mapping parameters were set as follows: QTL walking speed of 4 cM and stepwise regression probability of 0.001. Both GLM and MLM methods were tested, but GLM was found better fitting and generating more significant associations, which is not uncommon for panels with limited and clearly structured subpopulations, as it is the case for a NAM. So, the power of the NAM design was exploited to run genome-wide association analysis (GWAS) for all populations using a general linear model (GLM) with covariate parameter Q (population structure) in TASSEL v.5 (Bradbury et al., 2007), setting a kinship matrix with *k* = 3 and imposing DtH as covariate in all analyses to remove the strong effects of flowering genes from the study.

For both GWAS and QTL analysis, the threshold LOD for significance was set by the LD-protected Bonferroni method (Duggal et al., 2008; Bassi et al., 2019): 266 hypotheses of MTAs were investigated and calculated as the map size (1,879 cM) divided by the LD decay value (7.06 cM), which defined a significant threshold of LOD = 3.4 (*p* < 0.01). In addition, Pearson’s critical values (Pearson, 1985) defined *r* ^2 ^= 0.054 as the significance threshold for the minimum phenotypic variance explained by each marker. Only MTAs with LOD and *r* ^2^ superior to these threshold values were considered valid and presented here. Markers underlying MTA falling at less than twice the LD distance from each other were deemed impossible to distinguish genetically by the NAM panel and, hence, were declared as belonging to the same QTL.

### Validation studies in GWAS panel 2 and MAS panel 3

The three-step procedure to reduce type II errors is depicted in **Figure S1** and described hereafter using the NAM for QTL discovery, GWAS for allelic investigation, and the MAS panel for validation. Panel 2 GWAS was genotyped by the 35K Axiom breeder array as explained in detail in Kabbaj et al. (2017). The allelic investigation was conducted for the GWAS panel 2 to confirm which QTL identified in NAM panel 1 was additive in nature. Alignment between the QTLs identified in the 25K array of panel 1 NAM and Axiom 35K array was achieved using the relative physical positioning of marker probes on the Svevo assembly by *blast* with a similarity cutoff set at 99% (Maccaferri et al., 2019). Discrete classes of alleles were defined for the main MTA within each QTL associated with TKW. Genotypes within classes were defined as replicates (random), and their phenotypic performances under drought stress were tested in a two-way linear analysis of variance (ANOVA). The least significant difference (LSD) test was used to determine significantly superior classes of alleles using the LSD test function of the *agricolae* package (R Core Team, 2017; De Mendiburu and Yaseen, 2020).The 25K array markers underlying MTA associated with TKW were converted to KASP markers by submitting their array sequences to LGC Genomics for proprietary *in-silico* design. Those designs that passed the “success likelihood criteria” were purchased and used to genotype the MAS validation panel 3. The primer sequences cannot be publicly disclosed, but the markers are commercially available for use by providing the marker ID reported here. For this panel, the correlation significance threshold was calculated at *r* = 0.105 (*p* < 0.05) (Pearson, 1985). In addition, the top 20 and bottom 20 lines in terms of TKW performances were considered as the true positive and true negative. Hence, the accuracy was calculated as the ratio of the correct allelic call, sensitivity as the ratio of the correct positive allelic calls among the top 20 lines, and specificity as the ratio of the correct negative allelic calls among the bottom 20 lines.

## Results

### Moisture effect on traits and NAM genotypes’ responses

The regression analysis against the climate matrix identified the amount of moisture during the vegetative stage as the main factor influencing GY, while for TKW, it was the moisture amount during flowering and grain filling that were the most critical climatic factors (**Table S5**). Two clusters were obtained when grouping environments based on these three critical climatic factors: a moisture-stressed cluster of five environments (Syr rainfed 19 and 20, Leb rainfed 19 and 20, and Mor rainfed 20) and a second non-moisture-stressed cluster of three environments (Syr irrigated 19 and 20 and Leb irrigated 20) (**Figure S2**). For overall moisture, the lowest value was recorded at Mor rainfed 20 with just 209.5 mm, while the highest was Leb irrigated 20 season with 498.8 mm.

The descriptive statistics for the 430 NAM genotypes evaluated across the eight environments is reported in **Table S6**. The combined analysis of variance across eight environments shows that the effects of genotypes, environment, and their interactions were highly significant for all traits. TKW was the most heritable trait of the study (*H* ^2 ^= 0.94) and also GY had good values thanks to the NAM design (*H* ^2 ^= 0.52), while it was 0.42 for Spk.m^2^ and 0.65 for Grn.Spk and above 0.70 for all phenological traits (DTH, DTM, and GFR). Among the parents, the top yielder was Faraj with 3,221 kg ha^−1^ followed by Nachit, Jabal, and DAWRYT110 with 3,185, 3,172, and 3,121 kg ha^−1^, respectively. Pop1 (Nachit/DAWRYT110) recorded the highest average GY at 3,180 kg ha^−1^ and Pop3 (Nachit/Jabal) had the lowest at 3,158 kg ha^−1^. The GY performance of the tested genotypes across environments (BLUP) varied from 2,970 kg ha^−1^ for the lowest yielding progeny NAM-153 which belongs to Pop2 (Nachit/Faraj) to 3,269 kg ha^−1^ for the highest yielding progeny NAM-119 from Pop1 (Nachit/DAWRYT110).

**Figure 1:**
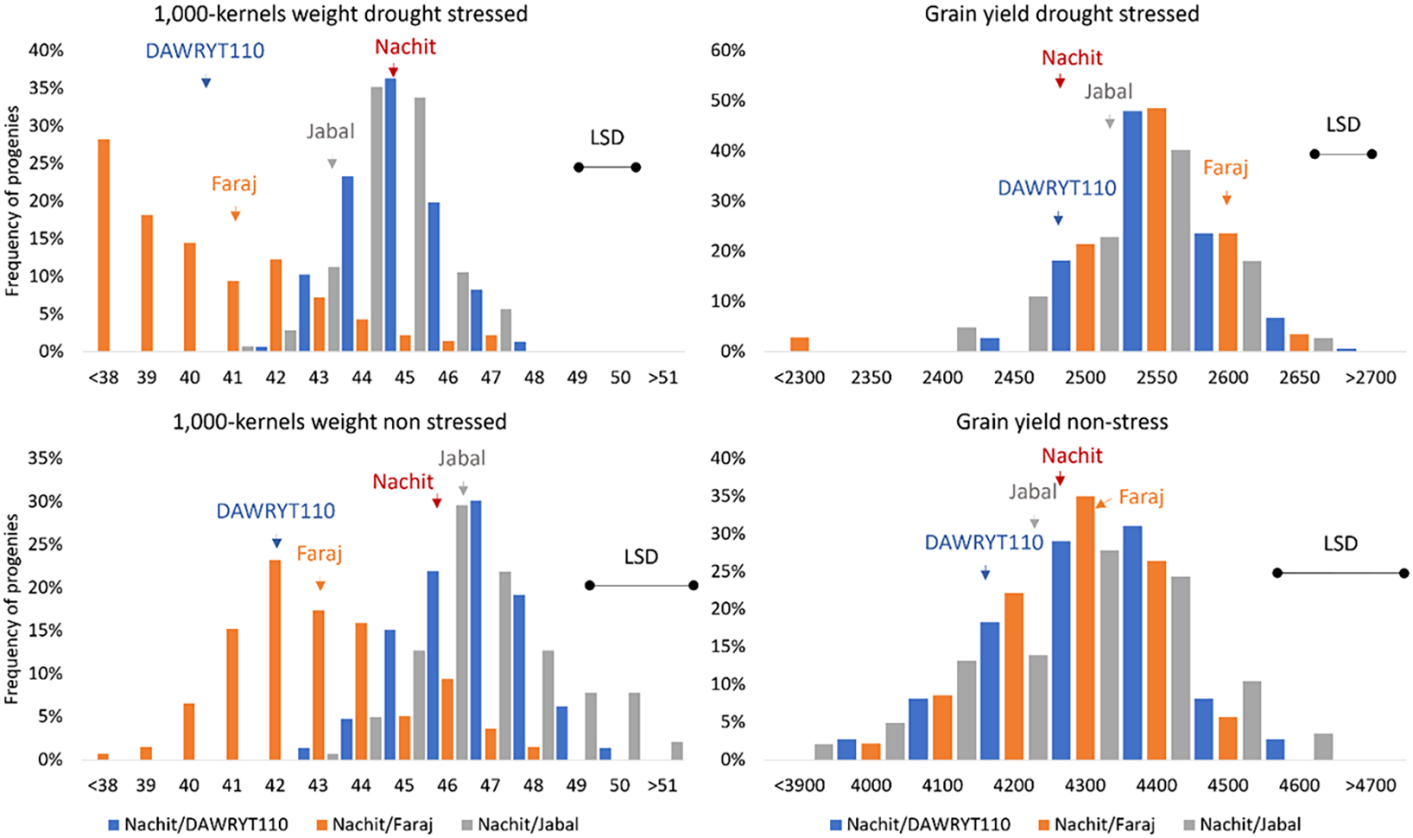
Distribution of performances of the NAM panel for 1,000 kernel weight (TKW, left side) and grain yield (GY, right side) presented as BLUP calculated across the drought-stressed cluster (top) and the non-stressed cluster (bottom). The populations are color-coded as per the parent naming (blue: pop1 Nachit/DWARYT110, orange: Pop2 Nachit/Faraj, gray: Pop3 Nachit/Jabal). The performances of the parents are presented as vertical arrows in the corresponding bin. The LSD for each experiment is presented as a horizontal black line to help determine significant differences among BLUPs.

The average value for TKW across environments was 43.5 g, with the parent Nachit having the highest score, followed by Jabal, Faraj, and DAWRYT110. Both Pop1 (Nachit/DAWRYT110) and Pop3 (Nachit/Jabal) reached an average of 45 g, while Pop2 (Nachit/Faraj) reached only 40 g. In fact, the top progeny for TKW was NAM-400 at 47.8 g from Pop3 (Nachit/Jabal) and the lowest was NAM-164 at 33.4 g from Pop2 (Nachit/Faraj) (**Figure 1**).

The two clusters of environments show diverse effects (**Table S7** and **Figure S3**): under non-stressed conditions, GY averaged 4,251 kg ha^−1^, while under moisture stress, it was 2,520 kg ha^−1^ (41% reduction). When assessing the parents’ performances within the two clusters, Faraj remained the top yielder with 2,566 and 4,347 kg ha^−1^ under moisture-stressed and non-stressed conditions, respectively. Within the moisture-stressed cluster, the progeny NAM-041 from Pop1 (Nachit/DWARYT110) recorded the highest GY at 2,670 kg ha^−1^ equal to a 4.8% increase over its recurrent parent (Nachit) and 7.5% over its donor parent (DAWRYT110). Within the non-stressed cluster, the progeny NAM-413 from Pop3 (Nachit/Jabal) was the top yielder at 4,588 kg ha^−1^ equal to a 7.1% increase over its recurrent parent (Nachit) and 7.9% over its donor parent (Jabal). Whereas, TKW witnessed an average 6% reduction between the two clusters, shifting from an average of 45 g under non-stressed conditions to 43 g when the stress occurred. Nachit was the top parent within the moisture stress cluster at 44.7 g, while Jabal was the best parent under non-stressed conditions at 45.7 g. Under both conditions, Nachit and Jabal were the top parents. Progeny NAM-120 from Pop1 (Nachit/DAWRYT110) was the top line at 47.5 g under moisture-stressed conditions equal to a 6.2% gain over the recurrent parent, while for the non-stressed cluster, the top genotypes all belonged to Pop3 (Nachit/Jabal).

AWAI identifies stable genotypes by measuring the size of their deviation from the AMMI axis, which represents the response to G×E factors. Nevertheless, stable genotypes might also result from overall low GY performances. For that reason, it is good practice to combine the GY genetic (G) potential measured as the performances across all tested environments (BLUP) and GY stability (G×E) by AWAI (**Figure S4**). The top parent for G and G×E was Nachit. Among the progenies, NAM-032, NAM-034, NAM-058, and NAM-060 all belonging to Pop1 (Nachit/DAWRYT110), NAM-248 and NAM-252 from Pop2 (Nachit/Faraj), and NAM-302 and NAM-342 from Pop3 (Nachit/Jabal) were the top performers.

### Interactions among traits under moisture-stressed and non-stressed conditions

Within the non-stress cluster, GY was positively influenced by PLH (*p* < 0.01), Spk.m^2^ (*p* < 0.01), and TKW (*p* < 0.05), while DtH (*p* < 0.01) and DtM (*p* < 0.01) had a negative effect (**Table 1**). Path coefficient analysis (**Table S8**) defines the trait priorities to better distinguish the effect: TKW was identified as the strongest influencing trait on GY (0.49), followed by PLH (0.41) and Spk.m^2^ (0.38). An indirect effect was recorded for the GFR since it influences GY through the determination of TKW (0.45). As expected, there was an inverse relationship between TKW and Grn.Spk (−0.23). Within the moisture stress cluster of environments, GY was strongly influenced (*p* < 0.01) by TKW, Grn.Spk, Spk.m^2^, PLH, and GFP, while it was negatively impacted by DtH (**Table 1**). Path coefficient analysis confirmed that TKW had the highest positive direct effect on GY (0.7), followed by Grn.Spk (0.69) and Spk.m^2^ (0.46). Also, GFR had the highest positive indirect effect on GY through TKW (0.62) (**Table S9**).

**Table 1:** Correlation between grain yield, yield components, and associated traits in stressed (below diagonal) and non-stressed (above diagonal) conditions.

Trait	GY	TKW	Gr_spk	Spk_m2	PLH	GFR	GFP	DTH	DTM
GY	–	0.1*	−0.02	0.51**	0.56**	0.06	0.08	−0.16*	−0.23**
TKW	0.26**	–	−0.46**	−0.07	−0.02	0.92**	0.23**	−0.23**	−0.10*
Gr_spk	0.42**	−0.38**	–	0.11*	0.10*	−0.4**	−0.15**	0.21**	0.16**
Spk_m2	0.33**	−0.09	−0.13*	–	0.35**	−0.03	−0.11*	0.07	−0.05
PLH	0.37**	0.46**	−0.04	0.32**	–	−0.01	−0.03	−0.04	−0.17**
GFR	0.08	0.88**	−0.39**	−0.25**	0.27**	–	−0.11	0.05	−0.09
GFP	0.15**	0.42**	−0.15*	−0.07	0.13*	0.13*	–	−0.86**	−0.1
DTH	−0.10*	−0.53**	0.17**	0.24**	−0.19**	−0.43**	−0.44**	–	0.56**
DTM	0.09	0.04	0.01	0.15*	0.03	−0.17**	0.46**	0.38**	–

GY, grain yield; TKW, 1,000-kernel weight; Gr_spk, grains per spike; Spk_m2, spikes per m^2^; PLH, plant height; GFR, grain filling rate; GFP, grain filling period; DTH, days to heading; DTM, days to maturity.

*Significant at the probability level 0.05.

**Significant at the probability level 0.01.

### Discovery of marker–trait associations in panel 1: NAM

**Figure 2:**
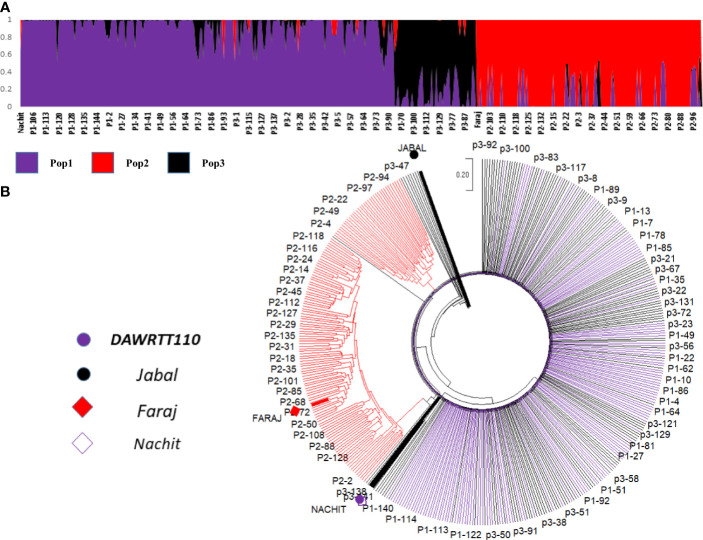
Population structure of three NAM populations (*k* = 3) shown as kinship. The *Y*-axis is the subpopulation relationship and the *X*-axis is the genotypes **(A)** and the phylogenetic tree based on the genetic distance calculated by neighbor-joining **(B)**. The progenies and parents are color-coded as per the legend.

The final consensus map of the NAM panel 1 incorporated 1,678 polymorphic markers with an average PIC of 0.28 grouped into 14 linkage groups spanning 1,723 cM. The largest chromosome was 7A consisting of 158 markers spanning 179 cM, and the smallest chromosome was 4B consisting of 38 markers covering 51 cM (**Table S10**). Imposing a kinship split with a *k* = 3 on the 426 RIL genotypes (**Figure 2A**) reidentified the three original subpopulations derived from different donor parents, confirming their relative genetic diversity, as well as the cluster analysis (**Figure 2B**) classified the 426 RIL genotypes into three groups based on genetic difference among their parents, and a genetic similarity was observed between the recurrent parent ‘Nachit’ and ‘DAWRYT110’ compared with ‘Jabal’ and ‘Faraj’.

Marker–trait association (MTA) analysis identified a total of 181 MTAs as meeting the minimum required threshold for LOD and *r* ^2^ considering all traits and conditions tested. Based on their genetic position, these could be merged into 18 QTLs appearing in two or more environments and identified by both QTL analysis and GWAS. These were numbered Q.ICD.NAM-01 to Q.ICD.NAM-18 (**Tables 2** and **S11**). Q.ICD.NAM-01 was identified on chromosome (chr) 1A (2.6–8 cM) at LOD of 7.4 associated with GY across two moisture-stressed environments and AWAI for TKW. It was also associated with PLH. Q.ICD.NAM-02 was identified on chr 1A (16–26.3 cM) at LOD of 9.7 associated with TKW in one moisture-stressed environment and GY in one moisture-stressed environment. Q.ICD.NAM-03 was identified at 48–50.12 cM on chr 1A (LOD = 11.4) explaining 5.7%–13.1% of the phenotypic variation (PV) for TKW across five environments, four of which were moisture-stressed, also co-associated with PLH, Grn.Spk, and AWAI for TKW. Q.ICD.NAM-04 was identified on the distal peri-telomeric region of chr 1B (LOD = 13.1) explaining 6% to 10.3% of the PV for TKW across five environments, four of which were moisture-stressed. This locus was also associated with GY across three moisture-stressed environments explaining 29.0%–47.8% PV and with AWAI for TKW. Q.ICD.NAM-05 was located on chromosome 2A (112.7–124 cM) at LOD of 9.7 and related to TKW across two moisture-stressed environments, GY in one moisture-stressed environment, and stability index AWAI for GY and PLH. Q.ICD.NAM-06 was identified on the distal peri-telomeric region of chromosome 2B (LOD = 8.9) explaining 28.7% to 46.5% PV for GY within the moisture-stressed cluster. This QTL was also associated with TKW in one moisture-stressed environment and with the stability index (AWAI) for GY. Q.ICD.NAM-07 was identified at 70.4–84 cM on chr 3A (LOD = 8.5) explaining 5.42%–6.04% of the PV for TKW across two moisture-stressed environments. This locus was also associated with AWAI for TKW and related with GY across one moisture-stressed and with PLH at one irrigated environment and across all environments. Q.ICD.NAM-08 is located between 36 cM and 48 cM on chr 3B (LOD = 9.3) explaining 5.8% to 44.7% of PV for TKW within the moisture-stressed cluster and 28.0% to 48.4% of the PV for GY in the same cluster of environments. It was also associated with Grn.Spk and with AWAI for GY. Q.ICD.NAM-09 was identified on the proximal peri-centromeric region of chr 4B (LOD = 6.8) explaining 28.5% to 46.4% of the PV for GY at two moisture-stressed environments. This QTL was also associated with PLH. Q.ICD.NAM-10 was located on chr 4B (30.5–40 cM) at LOD of 7.3 and associated with TKW across two moisture-stressed environments, GY in one moisture-stressed environment, and PLH. Q.ICD.NAM-11 was identified on the proximal peri-centromeric region of chr 5A (LOD = 11.1) explaining 49.6%–59.1% of the PV for GY across two moisture-stressed environments. This locus was also associated with TKW in one moisture-stressed environment and with PLH. Q.ICD.NAM-12 was identified on chr 5A (61.29–72 cM) at LOD of 7.6 and associated with GY across two moisture-stressed environments and with PLH across the combined irrigated cluster and across all environments. Q.ICD.NAM-13 was identified at 38.4–52 cM on chr 5B (LOD = 11.5) explaining 5.6%–8.4% of PV for TKW across four environments, three of which were moisture-stressed. It was also co-associated with GY under one moisture-stressed environment. Q.ICD.NAM-14 was identified at 60–72 cM on chr 6A (LOD = 9.1) explaining 5.2% to 7.2% of the PV for TKW across four environments, three of which were moisture-stressed. It also was co-associated with GY under one moisture-stressed environment and with PLH across environments. Q.ICD.NAM-15 was identified at 39.4–52 cM on chr 6B (LOD = 11.2) explaining 5.4%–31.4% of the PV for TKW across four moisture-stressed environments. It was also associated with GY across three moisture-stressed environments, Grn.Spk, and AWAI for GY. Q.ICD.NAM-16 was identified at 4–13.8 cM on chr 7A (LOD = 13.5) explaining 5.4–10.21 of the PV for TKW across six environments, five of which moisture-stressed. This QTL was also associated with GY across one moisture-stressed environments and PLH across environments. Q.ICD.NAM-17 was identified at 20–33.8 cM on chr 7A (LOD = 13.1) explaining 6.6%–13.1% of the PV for TKW across four environments, three of which were moisture-stressed. This locus was also associated with GY across two moisture-stressed environments explaining 28.2%–46% of its PV and with PLH across both moisture clusters. Q.ICD.NAM-18 was located at 36–50.0 cM on chr 7B (LOD = 13.2) explaining 5.8%–11.1% of the PV for TKW across six environments, five of which were moisture-stressed. It was also associated with GY in one moisture-stressed environment and AWAI for GY.

**Table 2:** Stable quantitative trait loci (QTLs) associated with multiple traits in a cluster of environments of stressed, non-stressed, and combined across all and the stability index (AWAI).

Locus	Chr	Trait	Main marker	Position cM	Max LOD	Max *r* ^2^	Stressed	Non-stressed	Combined	AWAI
1	1A	GY, PLH, AWAI	BS00011235_51	2.6–8.0	7.4	49.5	*	*	*	*
2	1A	GY, TKW	Tdurum_contig13459_543	0.0–10.3	9.7	46.3	*			
3	1A	TKW, Gr_spk, PLH, AWAI	AX-95079481	48.0–50.1	11.4	31.7	*	*		*
4	1B	TKW, GY, AWAI	Tdurum_contig12899_347	115.6–129.1	13.1	47.6	*	*		*
5	2A	TKW, GY, PLH, AWAI	Tdurum_contig13459_543	12.7–124.0	9.7	41.1	*	*	*	*
6	2B	GY, TKW, AWAI	AX-158567848	116.0–128.0	8.9	44.5	*		*	*
7	3A	GY, TKW, AWAI, PLH	Tdurum_contig86206_149	70.4–84.0	8.5	28.0	*	*	*	*
8	3B	GY, TKW, Gr_spk, AWAI	Kukri_c32046_493	36.0–48.0	9.3	48.4	*	*		*
9	4B	GY, PLH	BS00039936_51	16.0–24.0	6.8	46.3	*	*	*	
10	4B	GY, TKW, PLH	TGWA25K-TG0216	30.5–40.0	7.3	47.5	*	*	*	
11	5A	GY, TKW, PLH	AX-95200348	0.0–12.0	11.1	59.1	*	*	*	
12	5A	GY, PLH	wsnp_Ra_c17216_26044790	61.3–72.0	7.6	44.7	*	*	*	
13	5B	TKW, GY	AX-89660974	38.4–52.0	11.5	46.1	*	*		
14	6A	TKW, GY, PLH	AX-111052948	60.7	9.1	28.9	*	*	*	
15	6B	TKW, GY, Gr_spk	AX-109395546	39.4–52.0	11.2	46.0	*	*		
16	7A	TKW, GY, PLH	AX-158601039	4.0–13.8	13.5	19.3	*	*	*	
17	7A	GY, PLH, TKW	RAC875_rep_c111788_253	20–33.78	13.1	46.00	*	*		
18	7B	TKW, GY, AWAI	Tdurum_contig8448_363	36–49.96	13.2	42.08	*	*		*

GY, grain yield; TKW, 1,000-kernel weight; Gr_spk, grains per spike; PLH, plant height.

*Significant QTL.

### Effect of multiple QTLs for 1,000-kernels weight by allelic investigation: panels 1 and 2

The NAM panel was generated to segregate for TKW, the main trait of interest from the recurrent parent Nachit. Also, TKW showed a strong influence on GY under both moisture-stressed and non-stressed conditions. A total of 15 QTL associated with TKW including 14 co-associated with GY were investigated in NAM panel 1. Three QTLs (Q.ICD.NAM-04, Q.ICD.NAM-14, and Q.ICD.NAM-16) were confirmed by means of haplotype analysis when field-tested under drought conditions. To confirm that the effect of these loci was not spurious nor unique to the mapping populations under study, a second germplasm set named the “panel 2 GWAS” was investigated. This panel includes the parents of the NAM populations and several of their sister lines, in addition to other lines.

**Figure 3:**
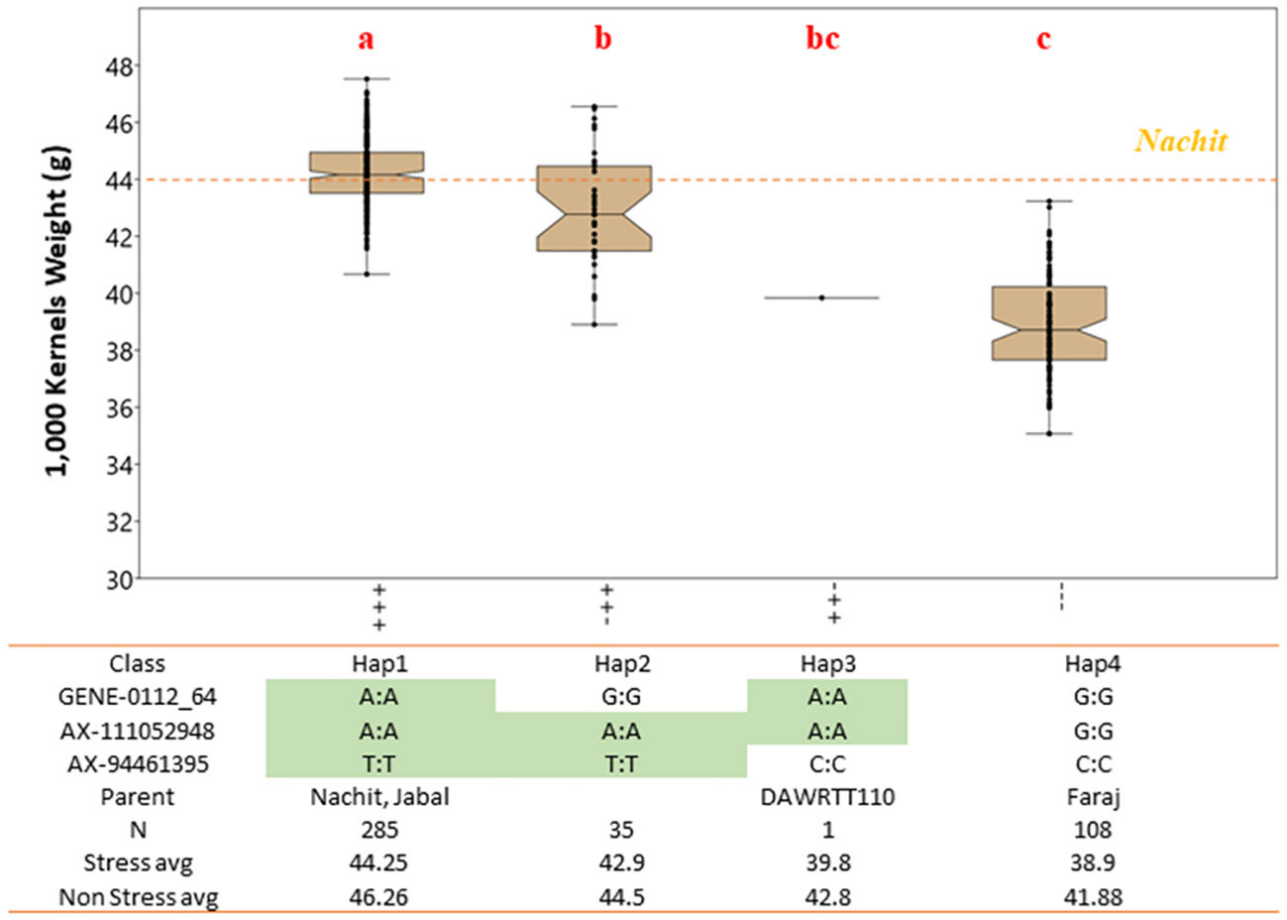
Effect of allelic combinations on 1,000-kernel weight for NAM accessions tested under drought-stressed conditions. The accessions were divided into four clusters based on their haplotype at three major QTLs. Letters above the whiskers indicate significant differences between the clusters.

**Figure 4:**
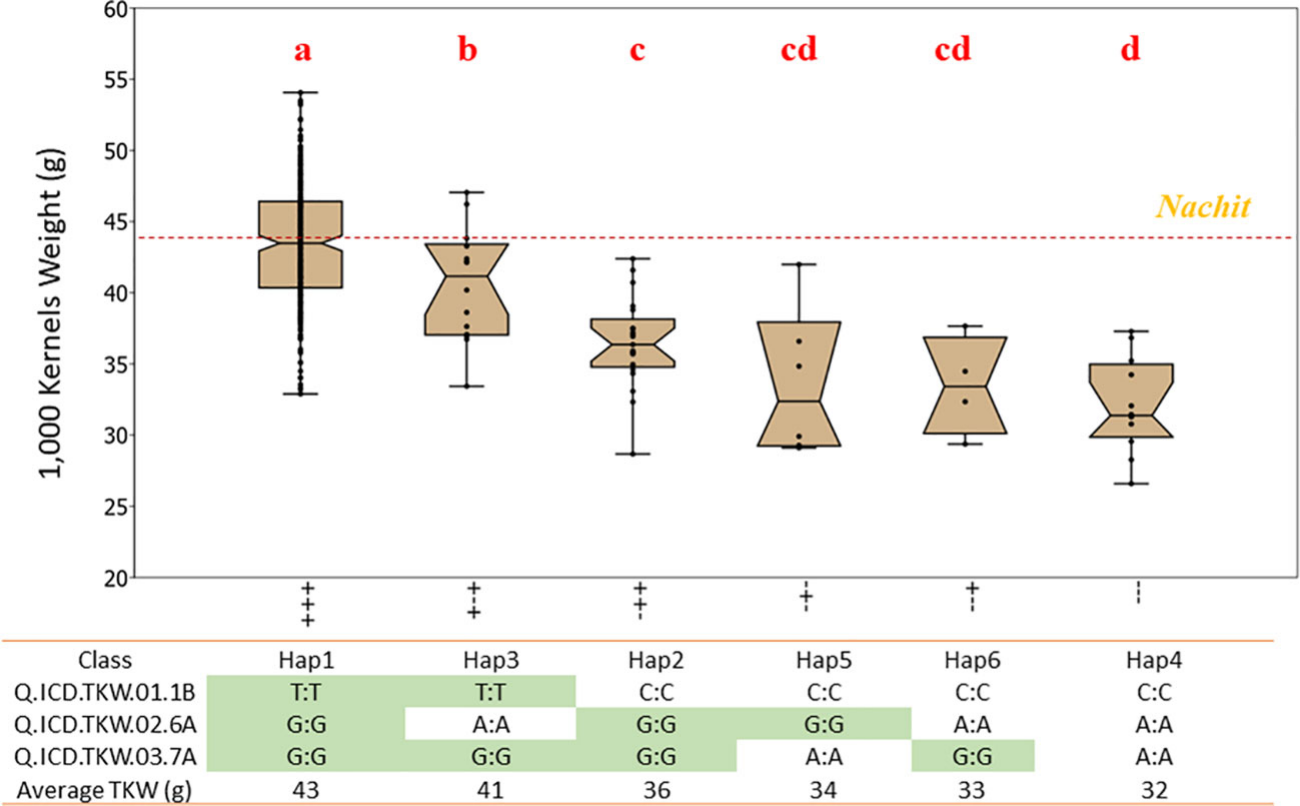
Effect of allelic combinations on 1,000-kernel weight for the “GWAS panel” tested under drought-stressed conditions. The accessions were divided into six clusters based on their haplotype for three major QTLs. Letters above the whiskers indicate significant differences between the clusters.

Within the NAM panel 1, a total of four allelic classes were identified, with haplotype 1 (Hap1), which carries a positive allele at each locus, achieving a significantly (*p* < 0.01) superior TKW of 44.2 g under moisture-stressed conditions, followed by Hap2 and Hap3 with only two positive alleles at 42.9 g and 39.8 g, respectively, and lastly Hap4 with the lowest TKW average of 38.9 g and carrying three negative alleles (**Figure 3**). The allelic study for the NAM confirmed the additive nature of these three QTLs achieving a 12.1% increase in TKW under drought stress. In addition, the allelic study confirmed that Nachit and Jabal both carry Hap1, while DAWRYT110 carries Hap3 and Faraj carries Hap4.The allelic investigation was carried out also in the panel 2 GWAS to confirm that six haplotypes existed in the broader germplasm set (**Figure 4**). Hap1 was confirmed as the significantly (*p* < 0.01) superior combination at an average TKW of 43 g across moisture-stressed environments, followed by Hap3 with positive alleles on chr 1B and 7A at 41 g, which in this case was significantly superior to Hap2 with positive alleles on chr 6A and 7A. The additional Hap5 and Hap6 carrying only one positive allele at chr 6A and 7A, respectively, were not significantly better than Hap4 which does not carry any positive alleles and reached an average TKW of 32 g. Hence, in this second panel, the three positive alleles resulted in an average increase of 25.6% for TKW under moisture stress.

### Validation of markers by KASP: panel 3

**Figure 5:**
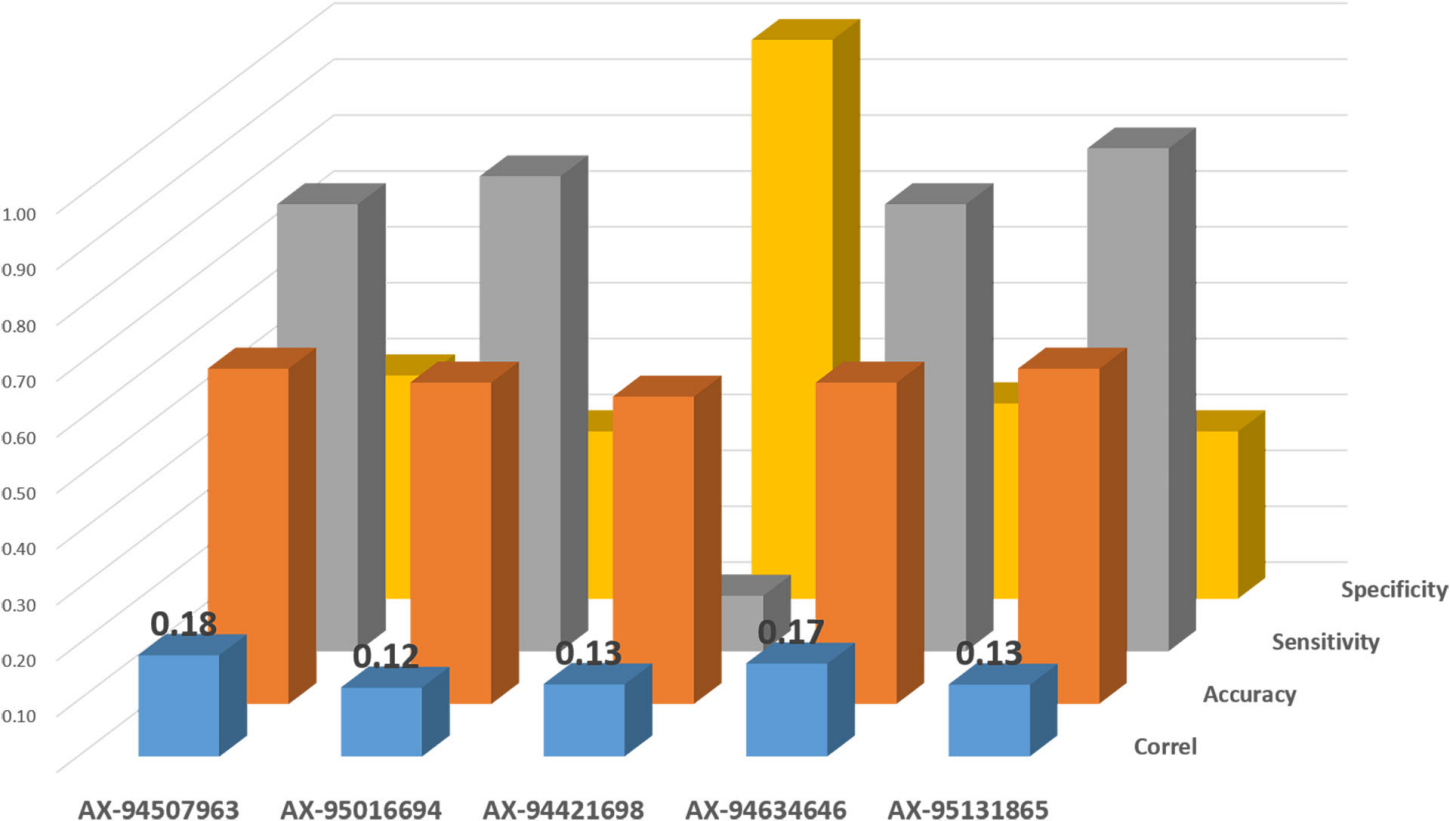
Validation of Kompetitive Allele-Specific PCR (KASP) markers on an independent set of elite ICARDA lines tested under drought stress for TKW.

“Validation” is a critical step required to convert QTL discovery into actual usable solutions for breeders by MAS or genomic selection. The conversion to KASP was attempted for those markers underlying the three QTLs investigated: Q.ICD.NAM-04, Q.ICD.NAM-14, and Q.ICD.NAM-16. A total of 10 KASP markers were used to genotype the validation set (**Figure 5**). Of these, five resulted as monomorphic in this germplasm panel, while five were polymorphic and passed the significance threshold for TKW under drought. AX-94507963 resulted as the most suitable to select for Q.ICD.NAM-04 with the highest correlation (*r* = 0.18) and accuracy (0.60). AX-94421698 was the only validated KASP for selecting the positive allele at Q.ICD.NAM-14 with a correlation of 0.13 and perfect specificity, while AX-94634646 was the most suitable to select the positive allele at Q.ICD.NAM-16 with 0.17 correlation and high sensitivity (0.80). Overall, only three of ICARDA’s elite lines (IDON43-56, IDON43-75, and IDON43-72) carried the positive allele at all three loci. These reached high TKW and carried *T. dicoccoides* in their pedigrees.

## Discussion

### Phenotypic performance of the NAM population

Drought stress is a major environmental factor limiting wheat production globally (Lascano et al., 2001; Daryanto et al., 2016; Negisho et al., 2022), and its effect affects grain yield and its components (Turner et al., 2014; Ayed et al., 2017; Khadka et al., 2020; Arriagada et al., 2022). In our study, drought stress caused a 41% loss in GY, which matches the values reported by Sukumaran et al. (2018) and Aberkane et al. (2020). However, the effect of drought stress on GY differs depending on the growth stage of occurrence, its duration, and its severity (Dehgahi et al., 2014; Semenov et al., 2014; González-Ribot et al., 2017; Sarto et al., 2017). Here, drought stress occurred primarily during the flowering and grain filling period, causing a 6% average reduction in TKW. Under both stressed and non-stressed conditions, TKW had a positive significant effect on GY, which was further confirmed by path analysis. This is in good agreement with other studies that also suggest TKW as an ideal trait to improve GY in durum wheat, especially to adapt to drought stress (García del Moral et al., 2003; Royo et al., 2014; González-Ribot et al., 2017). In fact, Nachit and several NAM progenies capable of achieving the highest TKW were also the top yielders and most stable. Furthermore, the high heritability and strong genetic control of TKW make it a trait of choice to achieve fast genetic improvement for tolerance to moisture stress (Xu et al., 2017). For that reason, further genetic dissection of this trait was sought.

### QTLs associated with grain yield and 1,000-kernel weight

Three subpopulations were identified based on structure and phylogenetic tree analysis, and the close genetic relation between the recurrent parent ‘Nachit’ and the donor parent ‘DAWRYT110’ was because they both have *T*. *dicoccoides* and Amedakul1 in their pedigree compared with the donor parent ‘Faraj’ which has *T. araraticum* in its pedigree and the same for the donor parent ‘Jabal’ which has *Ae. speltoides* in its pedigree.

The combined use of QTL analysis and GWAS applied to the NAM population discovered 18 QTLs on all chromosomes except 4A, 10 of them located on genome A and eight on genome B, 14 QTLs associated with TKW and GY, one QTL associated with TKW on chr 1A, and three QTLs associated with GY on chr 1A, 4B, and 5A. This fact highlights how the genetic contribution to TKW is highly related to the final GY performances under the studied conditions.

Chromosome 1A had three QTLs: Q.ICD.NAM-01 responsible for GY, Q.ICD.NAM-02 associated with both TKW and GY, and Q.ICD.NAM-03 linked to TKW. A similar region was previously identified by other studies under different water regimes for TKW (Ogbonnaya et al., 2017) and for GY (Tura et al., 2020; Zandipour et al., 2020). QTL Q.ICD.NAM-04 has been associated in this study with TKW across five environments and AWAI for TKW. A study by Maccaferri et al. (2015) involving 208 RILs derived from a cross between an elite durum cultivar and wild emmer also identified a stable QTL for TKW on chr 1B with a strong likely overlap with what was reported here. Similarly, Rehman Arif et al. (2020) reported two QTLs associated with TKW in the RIL population in the same region under drought stress. Fatiukha et al. (2020) identified stable QTLs for TKW on chromosomes 2A and 3A under five different environments using a population derived from *T. dicoccoides* which show a good overlap with our identified Q.ICD.NAM-05 and Q.ICD.NAM-07. Peleg et al. (2009) reported four stable QTLs for GY under both irrigated and water-limited conditions on chromosomes 2B, 4B, 4A, and 5A in a tetraploid wheat population derived from wild emmer (*T. dicoccoides*). In that study, QTLs overlapping with Q.ICD.NAM-06 on chr 2B and Q.ICD.NAM-11 on chr 5A positions were identified. Roncallo et al. (2017) reported three stable QTLs for GY across environments on chromosomes 3B and 2B using RIL durum wheat populations. Their reported QHi.cerz-2BS.1 shows a good overlap with our identified Q.ICD.NAM-06, and in both cases, these QTLs explained large PV.

Maccaferri et al. (2008) and Graziani et al. (2014) identified a stable QTL under both irrigated and rainfed conditions across eight water regime environments related to TKW and GY located on chromosome 3B using the RIL population, which shows a good overlap with our identified Q.ICD.NAM-08 also associated with GY and TKW across moisture-stressed environments. Zaïm et al. (2020) studied four RIL populations of durum wheat tested under drought stress conditions and identified six QTLs associated with GY. A likely overlap could be found for Q.ICD.NAM-06, Q.ICD.NAM-09, and Q.ICD.NAM-10. In addition, there were six individual QTLs for TKW located on chromosomes 1B, 4B, 6A, 6B, and 7A. A good overlap can be established by their reported Qicd.TKW.005 located on 6B and our identified Q.ICD.NAM-15 which was associated with TKW across four moisture-stressed environments and their Qicd.TKW.006 located on 7AS and our identified Q.ICD.NAM-16 responsible for the control of TKW across six environments. In particular, the QTL on chromosome 4B had also previously been confirmed as conferring drought tolerance in both bread wheat and durum wheat (Kadam et al., 2012; Wang et al., 2019; Rabbi et al., 2021).

Sukumaran et al. (2018) reported a strong association with TKW under yield potential, drought stress, and heat stress conditions for a region on chr 5B with a likely overlap to our reported Q.ICD.NAM-013. Similarly, Q.ICD.NAM-14 which controls TKW identified here also shows a likely overlap with a QTL reported by Avni et al. (2018) on the long arm of chr 6A named mQTL-GW-6A. In that study, a major candidate gene named “TtGRF4-A” was identified as associated with increased TKW. In the same region on 6A, Alahmad et al. (2019) reported a major QTL associated with seminal root angle (qSRA-6A) and TKW when assessing eight NAM durum wheat populations. Fatiukha et al. (2021) studied a RIL population derived from a cross between durum wheat and wild emmer to reveal two major QTLs associated with GY and TKW on chr 7B that show a likely overlap with our reported Q.ICD.NAM-18 which was associated with GY and TKW under drought conditions. In addition, they detected a QTL on chr 7A associated with TKW which overlaps with our reported Q.ICD.NAM-17 which is responsible for TKW across three moisture-stressed environments.

All the identified QTLs have been likely reported previously confirming the solidity of the study conducted. Out of 15 QTLs associated with TKW, three QTLs (Q.ICD.NAM.04, Q.ICD.NAM.14, and Q.ICD.NAM.16) on chromosomes 1B, 6A, and 7A appeared as the most critical for drought tolerance and associated with TKW and GY traits in three or more moisture-stressed environments and identified by both QTL analysis and GWAS. These three genomic regions were used to investigate the allelic combination responsible for TKW under drought conditions.

### Pyramiding drought-tolerant QTLs

Three QTLs (Q.ICD.NAM-04, Q.ICD.NAM-14, and Q.ICD.NAM-16) confirmed their additive nature by showing a 12.1% gain in TKW under drought stress for NAM lines carrying the positive allele at all three loci (Hap1). Furthermore, the same allelic investigation in a broader panel confirmed a positive gain of 25.6% for Hap1. Interestingly, the large-seeded varieties Nachit and Jabal both carry Hap1, which confirms the importance of these three QTLs. On the other hand, the 6% (+2.8 g) transgressive segregation for TKW demonstrated by NAM-120 (Nachit/DAWRYT110) carrying the Hap1 suggests that additional useful loci beyond these three might be available from DAWRYT110 (Hap3).

Nevertheless, one KASP was validated for each QTL with AX-94507963 tagging Q.ICD.NAM-04, AX-94421698 tagging Q.ICD.NAM-14, and AX-94634646 tagging Q.ICD.NAM-16. These three markers combined represent a ready resource for breeders to rapidly pyramid useful TKW starting from *T*. *dicoccoides* from the donor line Nachit. Because of Nachit’s origin from *T. dicoccoides*, because the only elites identified carrying the three alleles also include *T. dicoccoides* in their pedigrees, and because Maccaferri et al. (2015) reported Q.ICD.NAM-04 in a RIL study derived from *T. dicoccoides*, it is highly likely that the reported useful alleles are in fact originating from wild emmer. Nachit is currently undergoing whole genome sequencing and assembly, which once completed shall clarify the ancestral origin of the loci described here.

## Conclusions

To meet the future demand for durum wheat grains despite the climatic changes (Mulugeta et al., 2023), a significant increase in production is necessary (Sakuma and Schnurbusch, 2020). The indirect selection of production-related traits under more stringent genetic control is a potential solution to favor rapid pyramiding of useful alleles (Tadesse et al., 2019; Colasuonno et al., 2021). Larger 1,000-kernel weight is a demanded trait by the milling industry, thanks to its effect of increasing semolina yield, while it is also a production-related trait under strong genetic control and a major tolerance mechanism against terminal moisture stress. As such, the reported identification of useful alleles from recent durum wheat varieties should represent an opportunity for all breeders to rapidly improve TKW. For those breeders interested in accessing the donor parent Nachit (Taghouti et al., 2023), this is readily available as part of the Global Durum Panel (Mazzucotelli et al., 2020) as GDPv2-153 or GDPv1-152. Furthermore, the use of a three germplasm panel approach has allowed the identification and rapid validation of KASP markers that should significantly simplify the pyramiding of these alleles. The use of CWR-derived populations has likely contributed to the identification of novel alleles that were not previously available for durum wheat and that could be readily transferred also to bread wheat.

## Data availability statement

The germplasm described here is available through ICARDA’s genebank and can be requested here: https://www.genesys-pgr.org/wiews/SYR002. The genotypic and phenotypic data have been provided as **Supplementary Tables S1** and **S2** for panel 1 and 3, respectively. Phenotypic and genotypic data for panel 2 are freely available online as part of the article Zaïm et al., 2023.

## Author contributions

YJ: Data curation, Formal Analysis, Investigation, Visualization, Writing – original draft, Writing – review & editing. MH: Investigation, Writing – review & editing, Supervision. AA-Y: Methodology, Writing – review & editing, Validation. MS: Supervision, Writing – review & editing. AS: Conceptualization, Writing – review & editing. HK: Formal Analysis, Investigation, Methodology, Software, Writing – review & editing. MZ: Data curation, Formal Analysis, Investigation, Methodology, Software, Writing – review & editing. CK: Funding acquisition, Project administration, Resources, Supervision, Writing – review & editing. FB: Conceptualization, Funding acquisition, Investigation, Methodology, Project administration, Supervision, Writing – original draft, Writing – review & editing.

## References

[B1] Aberkane H. Amri A. Belkadi B. Filali-Maltouf A. Kehel Z. Tahir I. S. A.. (2020). Evaluation of durum wheat lines derived from interspecific crosses under drought and heat stress. Crop Sci. 61, 119–136. doi: 10.1002/csc2.20319

[B2] Aberkane H. Amri A. Belkadi B. Filali-Maltouf A. Kehel Z. Tahir I. S. Meheesi S.. (2021). Evaluation of durum wheat lines derived from interspecific crosses under drought and heat stress. Crop Sci. 61, 119–136. doi: 10.1002/csc2.20319

[B3] Alahmad S. El Hassouni K. Bassi F. M. Dinglasan E. Youssef C. Quarry G.. (2019). A major root architecture QTL responding to water limitation in durum wheat. Front. Plant Sci. 10, 436. doi: 10.3389/fpls.2019.00436 31024600 PMC6468307

[B4] Alahmad S. Kang Y. Dinglasan E. Jambuthenne D. Robinson H. Tao Y.. (2022). A multi-reference parent nested-association mapping population to dissect the genetics of quantitative traits in durum wheat. Genet. Resour. Crop Evol 70, 1471–1485. doi: 10.1007/s10722-022-01515-2

[B5] Alahmad S. Kang Y. Dinglasan E. Mazzucotelli E. Voss-Fels K. P. Able J. A.. (2020). Adaptive traits to improve durum wheat yield in drought and crown rot environments. Int. J. Mol. Sci. 21 (15), 5260. doi: 10.3390/ijms21155260 32722187 PMC7432628

[B6] Alvarado G. Lopez M. Vargas M. Pacheco A. Rodriguez F. Burgue˜no J.. (2015). META-R (Multi environment Trial Analysis with R for Windows). Version 5.0 (Mexico, D.F: CIMMYT).

[B7] Annual Agriculture Statistical Abstract (2020). Statistical Department, Office of Statistical, Ministry of Agriculture and Agrarian Reform (MOAAR), Damascus, Syria Arab Republic.

[B8] Arriagada O. Gadaleta A. Marcotuli I. Maccaferri M. Campana M. Reveco S.. (2022). A comprehensive meta-QTL analysis for yield-related traits of durum wheat (*Triticum turgidum* L. var. *durum*) grown under different water regimes. Front. Plant Sci. 13. doi: 10.3389/fpls.2022.984269 PMC9486101 36147234

[B9] Avni R. Oren l. Shabtay G. Assili S. Pozniak C. Hale I.. (2018). Genome based meta-QTL analysis of grain weight in tetraploid wheat identifies rare alleles of GRF4 associated with larger grains. Genes 9 (12), 636. doi: 10.3390/genes9120636 30562998 PMC6315823

[B10] Ayed S. Othmani A. Rezgui M. Ben Younes M. (2017). A review: Morphological, physiological, biochemical and molecular plant responses to water deficit stress. Int. J. Eng. Sci. 6, 1–4. doi: 10.9790/1813-0601010104

[B11] Babaiants O. V. Babayants L. T. Gorash A. F. Vasil’ev A. A. Traskovetskaya V. A. Palyasnyi V. A. (2012). Genetic determination of wheat resistance against *Puccinia graminis* (f. sp. *Tritici*) derived from *Aegilops cylindrica Triticum erebun*, and Amphidiploid 4. Cytol. Genet. 46, 10–17. doi: 10.3103/S0095452712010033 22420215

[B12] Bassi F. M. Brahmi H. Sabraoui A. Amri A. Nsarellah N. Nachit M. M.. (2019). Genetic identification of loci for Hessian fly resistance in durum wheat. Mol. Breeding. 24, 24–39. doi: 10.1007/s11032-019-0927-1

[B13] Bassi F. M. Nachit M. M. (2019). Genetic gain for yield and allelic diversity over 35 years of durum wheat breeding at ICARDA. Crop Breed. Genet. Genomics 1, 1–9. doi: 10.20900/cbgg20190004

[B14] Bassi F. M. Sanchez-Garcia M. (2017). Adaptation and stability analysis of ICARDA durum wheat (*Triticum durum* Desf.) elites across 18 countries. Crop Sci. 57, 1–12. doi: 10.2135/cropsci2016.11.0916

[B15] Bradbury P. J. Zhang Z. Kroon D. E. Casstevens T. M. Ramdoss Y. Buckler E. S. (2007). TASSEL: software for association mapping of complex traits in diverse samples. Bioinformatics 23, 2633–2635. doi: 10.1093/bioinformatics/btm308 17586829

[B16] Burton G. W. Devane E. H. (1953). Estimation of heritability in tall Festuca (*Festuca arudindcea*) from replicated clonal material. Agron. J. 45, 478–481. doi: 10.2134/agronj1953.00021962004500100005x

[B17] Chidzanga C. Mullan D. Roy S. Baumann U. Garcia M. (2022). Nested association mapping-based GWAS for grain yield and related traits in wheat grown under diverse Australian environments. Theor. Appl. Genet. 135 (12), 4437–4456. doi: 10.1007/s00122-022-04230-9 36205736 PMC9734238

[B18] Colasuonno P. Marcotuli I. Gadaleta A. Soriano J. M. (2021). From genetic maps to QTL cloning: an overview for durum wheat. Plants 10 (315), 1–25. doi: 10.3390/plants10020315 PMC7914919 33562160

[B19] Daryanto S. Wang L. Jacinthe P. A. (2016). Global synthesis of drought effects on maize and wheat production. PloS One 11 (5), e0156362. doi: 10.1371/journal.pone.0156362 27223810 PMC4880198

[B20] Dehgahi R. Joniyas A. Latip, S. N. H. B. M. D (2014). Rainfall distribution and temperature effects on wheat yield in Torbate Heydarie. Int. J. Sci. Res. Know. 2, 121–126.

[B21] De Mendiburu F. Yaseen M. (2020). Agricolae: statistical procedures for agricultural research. R Package version. 1.4. 0.

[B22] Djanaguiraman M. Prasad P. V. V. Kumari J. Sehgal S. K. Friebe B. Djalovic I.. (2019). Alien chromosome segment from *Aegilops speltoides* and Dasypyrum villosum increases drought tolerance in wheat via profuse and deep root system. BMC Plant Biol. 19, 242–315. doi: 10.1186/s12870-019-1833-8 31174465 PMC6554880

[B23] Duggal P. Gillanders E. M. Holmes T. N. Bailey-Wilson J. E. (2008). Establishing an adjusted p-value threshold to control the family-wide type 1 error in genome wide association studies. BMC Genomics 9, 516. doi: 10.1186/1471-2164-9-516 18976480 PMC2621212

[B24] El Haddad N. Kabbaj H. Zaïm M. El Hassouni K. Tidiane Sall A. Azouz M.. (2020). Crop wild relatives in durum wheat breeding: Drift or thrift? Crop Sci. 2020, 1–18. doi: 10.1002/csc2.20223

[B25] El Haddad N. Sanchez-Garcia M. Visioni A. Jilal A. El Amil R. Sall A. T.. (2021). Crop wild relatives crosses: multi-location assessment in durum wheat, barley, and lentil. Agronomy 11 (11), 2283. doi: 10.3390/agronomy11112283

[B26] El Hassouni K. Alahmad S. Belkadi B. Filali-Maltouf A. Hickey L. Bassi F. (2018). Root system architecture and its association with yield under different water regimes in durum wheat. Crop Sci. 58, 1–16. doi: 10.2135/cropsci2018.01.0076

[B27] El Hassouni K. Belkadi B. Filali-Maltouf A. Sall A. T. Al-Abdallat A. Nachit M.. (2019). Loci controlling adaptation to heat stress occurring at the reproductive stage in durum wheat. Agronomy 9, 414. doi: 10.3390/agronomy9080414

[B28] Fatiukha A. Deblieck M. Klymiuk V. Merchuk-Ovnat L. Peleg Z. Ordon F.. (2021). Genomic architecture of phenotypic plasticity in response to water stress in tetraploid wheat. International. J. Mol. Sci. 22 (4), 1723. doi: 10.3390/ijms22041723 PMC7915520 33572141

[B29] Fatiukha A. Filler N. Lupo I. Lidzbarsky G. Klymiuk V. Korol A. B.. (2020). Grain protein content and thousand kernel weight QTLs identified in a durumXwild emmer wheat mapping population tested in five environments. Theor. Appl. Genet. 133 (1), 119–131. doi: 10.1007/s00122-019-03444-8 31562566

[B30] Galili T. Dendextend (2015). An R package for visualizing, adjusting and comparing trees of hierarchical clustering. Bioinformatics 31, 3718–3720. doi: 10.1093/bioinformatics/btv428 26209431 PMC4817050

[B31] García del Moral L. F. Rharrabti Y. Villegas D. Royo C. (2003). Evaluation of grain yield and its components in durum wheat under mediterranean conditions: an ontogenic approach. Agron. J. 95, 266–274. doi: 10.2134/agronj2003.0266

[B32] González-Ribot G. Opazo M. Silva P. Acevedo E. (2017). Traits Explaining Durum Wheat (*Triticum turgidum* L. spp. *Durum*) Yield in Dry Chilean Mediterranean Environments. Front. Plant Sci. 8. doi: 10.3389/fpls.2017.01781 PMC5654942 29104578

[B33] Gonzalez-Segura E. Magaña-Barajas E. Torres-Chávez P. I. Manthey F. Ramírez-Wong B. (2014). Characterization of the dynamic viscoelastic behavior of semolina dough obtained from Mexican durum wheat cultivars. A. C.E.R. 3, 58–63.

[B34] Graziani M. Maccaferri M. Royo C. Salvatorelli F. Tuberosa R. (2014). QTL dissection of yield components and morpho-physiological traits in a durum wheat elite population tested in contrasting thermo-pluviometric conditions. Crop Pasture Sci. 65 (1), 80–95. doi: 10.1071/CP13349

[B35] Graziano S. Marmiroli N. Visioli G. Gullì M. (2020). Proteins and metabolites as indicators of flours quality and nutritional properties of two durum wheat varieties grown in different Italian locations. Foods 9, 315. doi: 10.3390/foods9030315 32182868 PMC7143883

[B36] Gupta P. Kabbaj H. El hassouni K. Maccaferri M. Sanchez-Garcia M. Tuberosa R.. (2020). Genomic regions associated with the control of flowering time in durum wheat. Plants 9, 1628. doi: 10.3390/plants9121628 33255147 PMC7759329

[B37] Jing R. Daokun S. Liang C. Frank M. Y. Jirui W. Yunliang P.. (2013). Genetic diversity revealed by single nucleotide polymorphism markers in a worldwide germplasm collection of durum wheat. Int. J. Mol. Sci. 14, 7061–7088. doi: 10.3390/ijms14047061 23538839 PMC3645677

[B38] Jujumaan (2017) Estimating and plotting the decay of linkage disequilibrium (Accessed 15 July 2017). jujumaan.com/2017/07/15/linkagedisequilibrium-decay-plot/.

[B39] Kabbaj H. Sall A. T. Al-Abdallat A. Geleta M. Amri A. Filali-Maltouf A.. (2017). genetic diversity within a global panel of durum wheat (*Triticum durum*) landraces and modern germplasm reveals the history of allele exchange. Front. Plant Sci. 8. doi: 10.3389/fpls.2017.01277 PMC5513985 28769970

[B40] Kadam S. Singh K. Shukla S. Goel S. Vikram P. Pawar V.. (2012). Genomic associations for drought tolerance on the short arm of wheat chromosome 4B. Funct. Integr. Genomics 12, 447–464. doi: 10.1007/s10142-012-0276-1 22476619

[B41] Khadka K. Earl H. J. Raizada M. N. Navabi A. (2020). A physio-morphological trait-based approach for breeding drought tolerant wheat. Front. Plant Sci. 11. doi: 10.3389/fpls.2020.00715 PMC7286286 32582249

[B42] Kiani R. Arzani A. Habibi F. (2015). Physiology of salinity tolerance in *Aegilops cylindrica* . Acta Physiol. Plant 37, 135–145. doi: 10.1007/s11738-015-1881-0

[B43] Kumar S. Stecher G. Li M. Knyaz C. Tamura K. (2018). MEGA X: Molecular Evolutionary Genetics Analysis across computing platforms. Mol. Biol. Evol. 35, 1547–1549. doi: 10.1093/molbev/msy096 29722887 PMC5967553

[B44] Lascano H. R. Antonicelli G. E. Luna C. M. Antonicelli G. E. Luna C. M. Melchiorre M. N.. (2001). Antioxidant system response of different wheat cultivars under drought: field and *in vitro* studies. Funct. Plant Biol. 28, 1095–1102. doi: 10.1071/PP01061

[B45] Ledesma-Ramírez L. Solís-Moya E. Iturriaga G. Sehgal D. Reyes-Valdes M. H. Montero-Tavera V.. (2019). GWAS to identify genetic loci for resistance to yellow rust in wheat pre-breeding lines derived from diverse exotic crosses. Front. Plant Sci. 10. doi: 10.3389/fpls.2019.01390 PMC6831551 31781137

[B46] Liu K. Muse S. V. (2005). Power Marker: An integrated analysis environment for genetic marker analysis. Bioinformatics 21, 2128–2129. doi: 10.1093/bioinformatics/bti282 15705655

[B47] Maccaferri M. Harris N. S. Twardziok S. O. Pasam R. K. Gundlach H. Spannagl M.. (2019). Durum wheat genome highlights past domestication signatures and future improvement targets. Nat. Genet. 51, 885–895. doi: 10.1038/s41588-019-0381-3 30962619

[B48] Maccaferri M. Ricci A. Salvi S. Milner S. G. Noli E. Martelli P. L.. (2015). A high-density, SNP based consensus map of tetraploid wheat as a bridge to integrate durum and bread wheat genomics and breeding. Plant Biotechnol. J. 13, 648–663. doi: 10.1111/pbi.12288 25424506

[B49] Maccaferri M. Sanguineti M. C. Corneti S. Ortega J. L. Salem M. B. Ben B.. (2008). Quantitative trait loci for grain yield and adaptation of durum wheat (Triticum durum desf.) across a wide range of water availability. Genetics 178 (1), 489–511. doi: 10.1534/genetics.107.077297 18202390 PMC2206097

[B50] Makai S. Tamás L. Juhász A. (2016). A catalog of regulatory sequences for trait gene for the genome editing of wheat. Front. Plant Sci. 7. doi: 10.3389/fpls.2016.01504 PMC5052276 27766102

[B51] Marais G. F. Badenhorst P. E. Eksteen. A. Pretorius Z. A. (2010). Reduction of *Aegilops sharonensis* chromatin associated with resistance genes Lr56 and Yr38 in wheat. Euphytica 171, 15–22. doi: 10.1007/s10681-009-9973-9

[B52] Marais G. F. Pretorius Z. A. Wellings C. R. McCallum B. Marais A. S. (2005). Leaf rust and stripe rust resistance genes transferred to common wheat from *Triticum dicoccoides* . Euphytica 143, 115–123. doi: 10.1007/s10681-005-2911-6

[B53] Masoomi-Aladizgeh F. Aalami A. Esfahani M. Aghaei M. J. Mozaffari K. (2015). Identification of CBF14 and NAC2 genes in *Aegilops tauschii* associated with resistance to freezing stress. Appl. Biochem. Biotech. 176, 1059–1070. doi: 10.1007/s12010-015-1629-80 25900437

[B54] Mazzucotelli E. Sciara G. Mastrangelo A. M. Desiderio F. Xu S. S. Faris J.. (2020). The global durum wheat panel (gdp): an international platform to identify and exchange beneficial alleles. Front. Plant Sci. 11. doi: 10.3389/fpls.2020.569905 PMC7779600 33408724

[B55] Meng L. Li H. H. Zhang L. Y. Wang J. K. (2015). QTL IciMapping: integrated software for genetic linkage map construction and quantitative trait locus mapping in biparental populations. Crop 3, 269–283. doi: 10.1016/j.cj.2015.01.001

[B56] Mulugeta B. Tesfaye K. Ortiz R. Johansson E. Hailesilassie T. Hammenhag C.. (2023). Marker-trait association analyses revealed major novel QTLs for grain yield and related traits in durum wheat. Front. Plant Sci. 13. doi: 10.3389/fpls.2022.1009244 PMC9909559 36777537

[B57] Negisho K. Shibru S. Matros A. Pillen K. Ordon F. Wehner G. (2022). Genomic dissection reveals QTLs for grain biomass and correlated traits under drought stress in Ethiopian durum wheat (*Triticum turgidum* ssp. *durum*). Plant Breed. 141 (3), 338–354. doi: 10.1111/pbr.13010

[B58] Nei M. Tajima F. Tateno Y. (1983). Accuracy of estimated phylogenetic trees from molecular data II. Gene frequency data. Theor. Appl. Genet. 19, 153–170. doi: 10.1007/BF02300753 6571220

[B59] Ogbonnaya F. C. Rasheed A. Okechukwu E. C. Jighly A. Makdis F. Wuletaw T.. (2017). Genome-wide association study for agronomic and physiological traits in spring wheat evaluated in a range of heat prone environments. Theor. Appl. Genet. 130, 1819–1835. doi: 10.1007/s00122-017-2927-z 28577083

[B60] Palta J. A. Turner N. C. (2018). Crop root system traits cannot be seen as a silver bullet delivering drought resistance. Plant Soil. 1, 31–43. doi: 10.1007/s11104-018-3864-6

[B61] Pearson K. (1985). Notes on regression and inheritance in the case of two parents. Proc. R Soc. Lond. 58, 240–242.

[B62] Peleg Z. V. I. Fahima T. Krugman T. Abbo S. Yakir D. A. N. Korol A. B.. (2009). Genomic dissection of drought resistance in durum wheat× wild emmer wheat recombinant inbreed line population. Plant Cell Environ. 32, 758–779. doi: 10.1111/j.1365-3040.2009.01956.x 19220786

[B63] Pritchard J. K. Stephens M. Donnelly P. (2000). Inference of population structure using multilocus genotype data. Genetics 155, 945–959. doi: 10.1093/genetics/155.2.945 10835412 PMC1461096

[B64] R Core Team (2017). R: A language and environment for statistical computing. (Vienna: R Foundation for Statistical Computing). Available at: https://www.R-project.org/.

[B65] Rabbi S. M. H. A. Kumar A. Mohajeri Naraghi S. Simsek S. Sapkota S. Solanki S.. (2021). Genome-wide association mapping for yield and related traits under drought stressed and non-stressed environments in wheat. Front. Genet 12. doi: 10.3389/fgene.2021.649988 PMC8258415 34239537

[B66] Rehman Arif M. A. Attaria F. Shokat S. Akram S. Waheed M. Q. Arif A.. (2020). Mapping of QTLs associated with yield and yield related traits in durum wheat (*Triticum durum* desf.) under irrigated and drought conditions. Int. J. Mol. Sci. 21, 2372. doi: 10.3390/ijms21072372 32235556 PMC7177892

[B67] Reynolds M. P. Pask A. J. D. Hoppitt W. J. E. Sonder K. Sukumaran S. Molero G.. (2018). Correction to: strategic crossing of biomass and harvest index-source and sink-achieves genetic gains in wheat. Euphytica 214 (1), 9. doi: 10.1007/s10681-017-2086-y 31187787 PMC6445510

[B68] Roncallo P. F. Akkiraju P. C. Cervigni G. L. Echenique V. C. (2017). QTL mapping and analysis of epistatic interactions for grain yield and yield-related traits in *Triticum turgidum* L. var. *durum* . Euphytica 213, 277. doi: 10.1007/s10681-017-2058-2

[B69] Royo C. Nazco R. Villegas D. (2014). The climate of the zone of origin of Mediterranean durum wheat (*Triticum durum* Desf.) landraces affects their agronomic performance. Genet. Resour. Crop Evol. 61, 1345–1358. doi: 10.1007/s10722-014-0116-3

[B70] Sakuma S. Schnurbusch T. (2020). Of floral fortune: Tinkering with the grain yield potential of cereal crops. New Phytol. 225, 1873–1882. doi: 10.1111/nph.16189 31509613

[B71] Sall A. T. Chiari T. Legesse W. Seid-Ahmed K. Ortiz R. van Ginkel M.. (2019). Durum wheat (*Triticum durum* desf.): origin, cultivation and potential expansion in sub-saharan Africa. Agron 9, 263. doi: 10.3390/agronomy9050263

[B72] Sall A. T. Cisse M. Gueye H. Kabbaj. H. Ndoyel I. Filali-Maltouf A.. (2018). Heat tolerance of durum wheat (*Tritcum durum* Desf.) elite germplasm tested along the Senegal River. J. Agric. Sci. 10, 217–233. doi: 10.5539/jas.v10n2p217

[B73] Sarto M. V. M. Sarto J. R. W. Rampim L. Bassegio D. da Costa P. F. Inagaki A. M. (2017). Wheat phenology and yield under drought: a review. Aust. J. Crop Sci. 11, 941–946. doi: 10.21475/ajcs17.11.08.pne351

[B74] Scott M. F. Ladejobi O. Amer S. Bentley A. R. Biernaskie J. Boden S. A.. (2020). Multi-parent populations in crops: a toolbox integrating genomics and genetic mapping with breeding. Heredity 125, 396–416. doi: 10.1038/s41437-020-0336- 32616877 PMC7784848

[B75] Semenov M. A. Stratonovitch P. Alghabari F. Gooding M. J. (2014). Adapting wheat in Europe for climate change. J. Cereal Sci. 59, 245–256. doi: 10.1016/j.jcs.2014.01.006 24882934 PMC4026126

[B76] Slafer G. A. Araus J. L. Royo C. García Del Moral L. F. (2005). Promising eco-physiological traits for genetic improvement of cereal yields in Mediterranean environments. Ann. Appl. Biol. 146, 61–70. doi: 10.1111/j.1744-7348.2005.04048.x

[B77] Sukumaran S. Reynolds M. P. Sansaloni C. (2018). Genome-wide association analyses identify QTL hotspots for yield and component traits in durum wheat grown under yield potential, drought, and heat stress environments. Front. Plant Sci. 9. doi: 10.3389/fpls.2018.00081 PMC5808252 29467776

[B78] Tadesse W. Suleiman S. Tahir I. Sanchez-Garcia M. Jighly A. Hagras A.. (2019). Heat-tolerant QTLS associated with grain yield and its components in spring bread wheat under heat-stressed environments of Sudan and Egypt. Crop Sci. 59, 199–211. doi: 10.2135/cropsci2018.06.0389

[B79] Taghouti M. Bassi F. M. Nasrellah N. Amri A. Motawaj J. Nachit M. (2023). ‘Nachit’, a wild-relative-derived durum wheat resilient to climate change in Morocco. J. Plant Registrations. 17, 529–535. doi: 10.1002/plr2.2029

[B80] Tura H. Edwards J. Gahlaut V. Garcia M. Sznajder B. Baumann U.. (2020). QTL analysis and fine mapping of a QTL for yield-related traits in wheat grown in dry and hot environments. Theor. Appl. Genet. 133, 239–257. doi: 10.1007/s00122-019-03454-6 31586227 PMC7990757

[B81] Turner N. C. Blum A. Cakir M. Steduto P. Tuberosa R. Young N. (2014). Strategies to increase the yield and yield stability of crops under drought—are we making progress? Funct. Plant Biol. 41 (11), 1199–1206. doi: 10.1071/FP14057 32481069

[B82] United States Department of Agriculture, Foreign Agricultural Service. (2022). Available at: https://www.fas.usda.gov/data/Morocco-durum-wheat-tender (Accessed 22September, 2022).

[B83] Van Oosten M. J. Costa A. Punzo P. Landi S. Ruggiero A. Batelli G.. (2016). “Genetics of drought stress tolerance in crop plants,” in Drought stress tolerance in plants. Eds. Hossain M. A. Wani S. H. Bhattachajee S. Burritt D. J. Tran L.-S. P. (Cham, Switzerland: Springer), 39–70.

[B84] Wang S. Xu S. Chao S. Sun Q. Liu S. Xia G. (2019). A genome-wide association study of highly heritable agronomic traits in durum wheat. Front. Plant Sci. 10. doi: 10.3389/fpls.2019.00919 PMC6652809 31379901

[B85] Xu Y. F. Li S. S. Li L. H. Ma F. F. Fu X. Y. Shi Z. L.. (2017). QTL mapping for yield and photosynthetic related traits under different water regimes in wheat. Mol. Breed. 37, 34. doi: 10.1007/s11032-016-0583-7

[B86] Xynias I. N. Mylonas I. Korpetis E. G. Ninou E. Tsaballa A. Avdikos I. D.. (2020). Durum wheat breeding in the mediterranean region: current status and future prospects. Agronomy 10, 432. doi: 10.3390/agronomy10030432

[B87] Zaïm M. Kabbaj H. Kehel Z. Gorjanc G. Filali-Maltouf A. Belkadi B.. (2020). Combining QTL analysis and genomic predictions for four durum wheat populations under drought conditions. Front. Genet. 11. doi: 10.3389/fgene.2020.00316 PMC7218065 32435259

[B88] Zaïm M. Sanchez-Garcia M. Belkadi B. Filali-Maltouf A. Al Abdallat A. Kehel Z.. (2023). Genomic regions of durum wheat involved in water productivity. J. Exp. Bot. 13, erad357. doi: 10.1093/jxb/erad357 PMC10735558 37702385

[B89] Zandipour M. Majidi Hervan E. Azadi A. Khosroshahli M. Etminan A. (2020). A QTL hot spot region on chromosome 1B for nine important traits under terminal drought stress conditions in wheat. Cereal Res. Commun. 48, 17–24. doi: 10.1007/s42976-020-00017-0

[B90] Zhang H. Mittal N. Leamy L. J. Barazani O. Song B. H. (2016). Back into the wild—Apply untapped genetic diversity of wild relatives for crop improvement. Evol. Appl. 10, 5–24. doi: 10.1111/eva.12434 28035232 PMC5192947

