# Supplementary material for: Genomic regions involved in the control of 1,000-kernel weight in wild relative-derived populations of durum wheat
Source: Front Plant Sci. 2023 Nov 30;14:1297131. doi: 10.3389/fpls.2023.1297131 (PMC10720367; doi:10.3389/fpls.2023.1297131)
Supplement: Supplementary file 1 [file DataSheet_1.pdf]

Supplementary Figures

Genomic Regions Involved in the Control of 1,000-kernels Weight in Wild Relatives-Derived Populations of Durum Wheat

Yaman Jabbour[1,2], Mohammad Shafik Hakim[1], Abdallah Al-Yossef[2], Maysoun M. Saleh[2], Ahmad Shams Al-Dien Shaaban[3], Hafssa Kabbaj[4], Meryem Zaïm[4], Charles Kleinerman[4], and Filippo M. Bassi[4]*

1 Field Crop Department, Faculty of Agriculture Engineering, Aleppo University/Syria
2 General commission for scientific Agriculture research (GCSAR) / Syria.
3 Biotech Engineering Department, Faculty of Technological Engineering, Aleppo University/Syria
4 ICARDA, Biodiversity and Integrated Gene Management, Rabat, Morocco.
*** Correspondence:** Corresponding Author: Filippo M. Bassi: f.bassi@cgiar.org

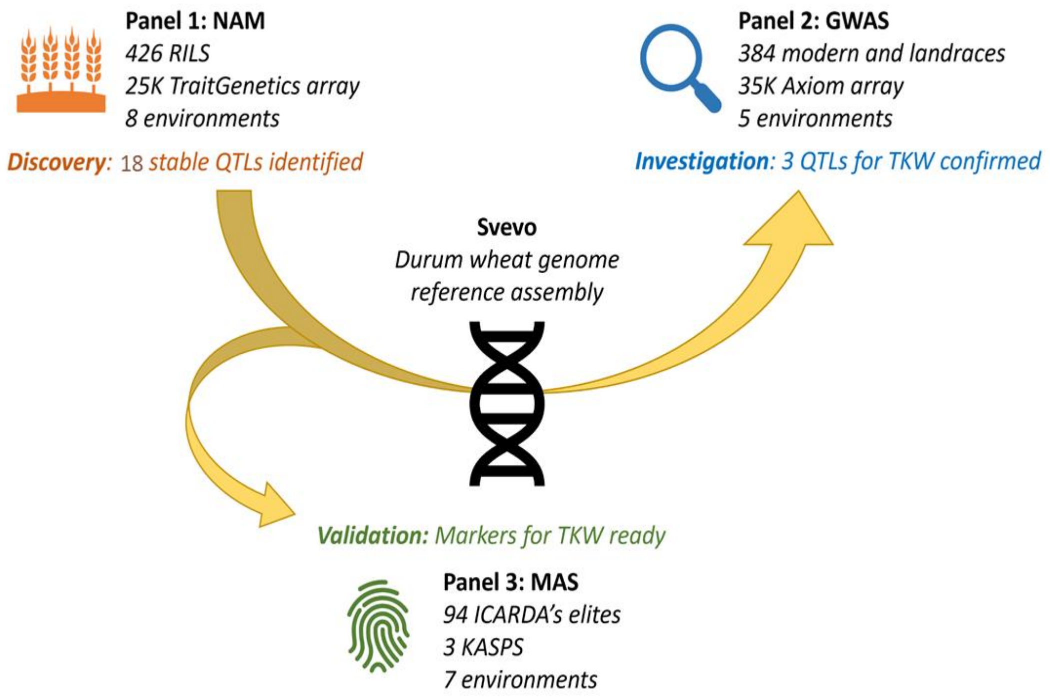

Figure S1 Graphical representation of the three steps validation approach. The NAM panel 1 is used for the discovery of QTL, then the GWAS panel 2 is used to investigate haplotype additive effects, and finally the MAS panel 3 is used to validate KASP. The Svevo genome assembly is used as the support for this approach, comparing the same physical regions across different genotyping protocols.

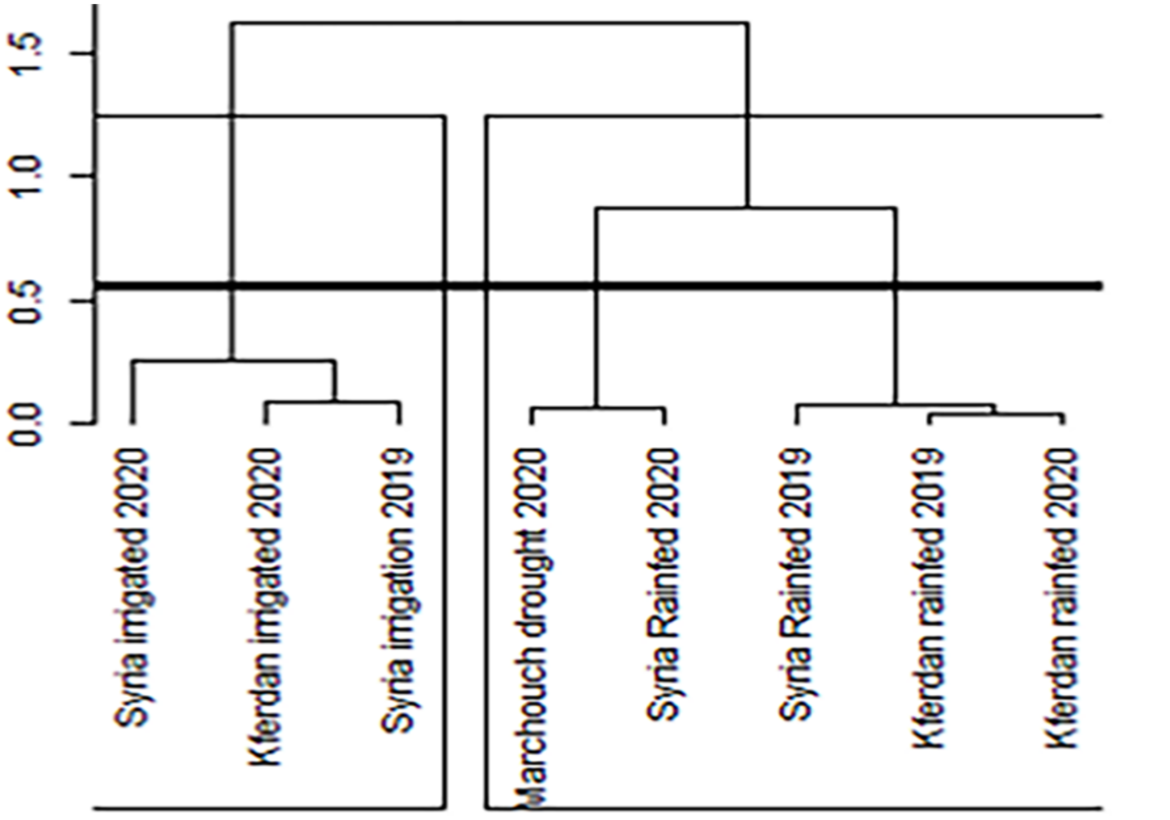

Figure S2 Cluster of Environments

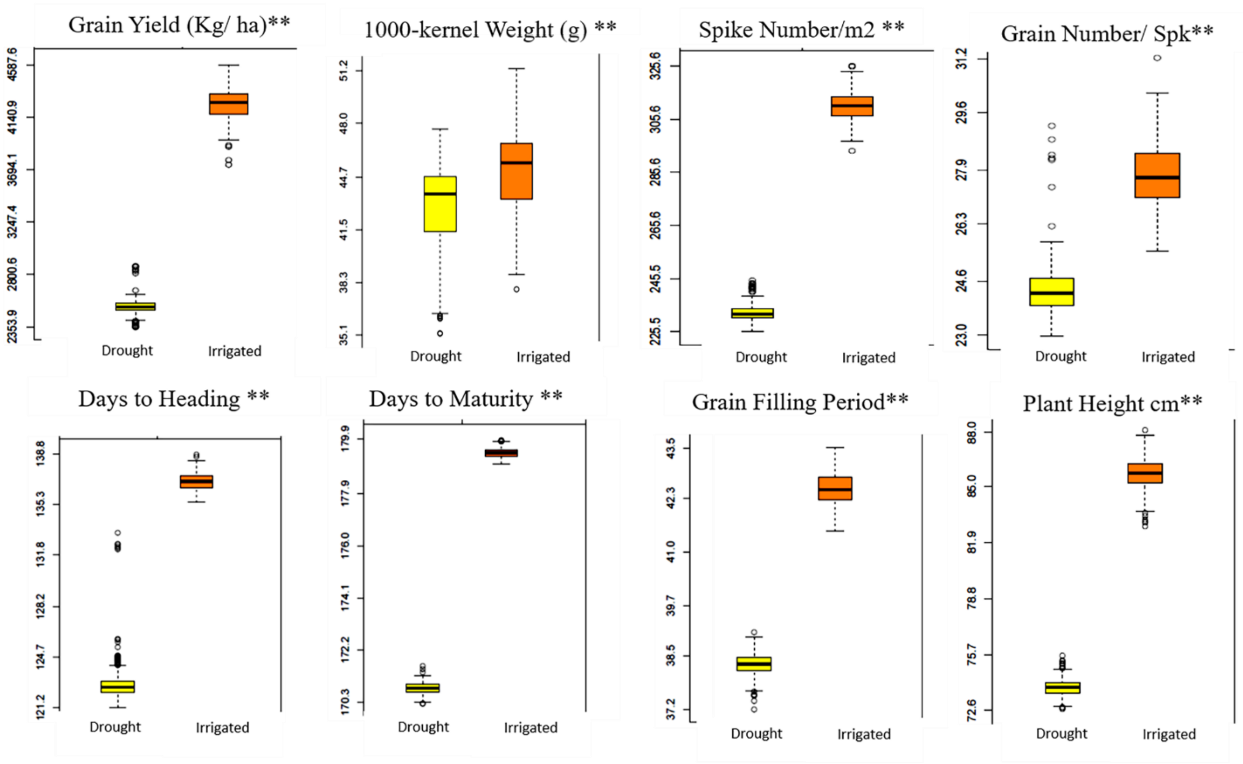

Figure S3 Boxplot of the best linear unbiased Predictors (Blups) for various traits under two different environmental conditions (Drought and irrigated)

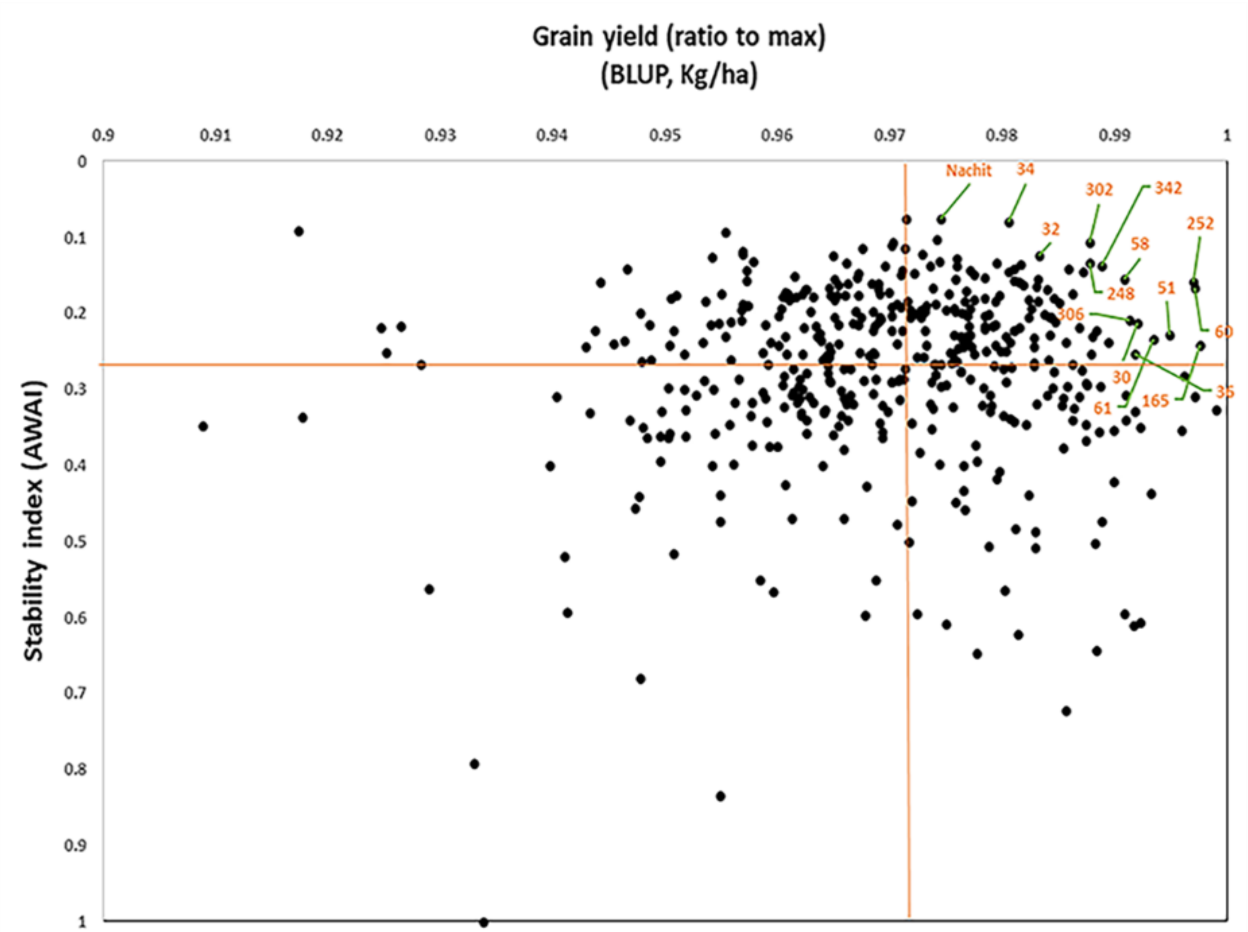

Figure S4 Yield stability response to GxE measured as AMMI wide adaptation index (AWAI) vs. grain yield potential (G) measured as BLUP across environments. The overall best entries are in the top right corner.
